# Seaweed-derived bioactives with anti-tyrosinase activity: a potential for skin-whitening cosmetics with *in silico* and *in vitro* approaches

**DOI:** 10.1016/j.btre.2025.e00910

**Published:** 2025-08-07

**Authors:** Arachaporn Thong-olran, Supatchar Sermsakulwat, Tiwtawat Napiroon, Phuphiphat Jaikaew, Sumet Kongkiatpaiboon, Ngampuk Tayana, Bongkot Wichachucherd, Theppanya Charoenrat, Thrissawan Traijitt, Supenya Chittapun

**Affiliations:** a Department of Biotechnology, Faculty of Science and Technology, Thammasat University, Rangsit Center, Pathum Thani 12121, Thailand; b Algae and Plankton Research Unit, Thammasat University, Rangsit Center, Pathum Thani, 12120, Thailand; c Drug Discovery and Development Center, Thammasat University, Rangsit Center, Pathum Thani 12121, Thailand; d Department of Science and Bioinnovation, Faculty of Liberal Arts and Science, Kasetsart University, Kamphang Sean, Nakorn Pathom 73140, Thailand

**Keywords:** Seaweed extract, Tyrosinase inhibitor, Hyperpigmentation, Stigmasterol, Skincare bioactive

## Abstract

Seaweed is a promising source of bioactive compounds with potential applications in skincare, particularly for addressing hyperpigmentation. This study investigated the chemical profiles and biological activities of hydrophilic and lipophilic extracts from *Sargassum polycystum, Caulerpa lentillifera*, and *Gracilaria fisheri*. LC-MS and GC–MS analyses identified 8 and 112 compounds in hydrophilic and lipophilic extracts, respectively. Among these, *G. fisheri* lipophilic extracts exhibited the strongest anti-tyrosinase activity. Molecular docking identified eight compounds with low binding affinities comparable to kojic acid and tropolone, while ADMET predictions highlighted stigmasterol for its favorable skin permeability and safety. *In vitro* cytotoxicity assays confirmed its low toxicity (IC_50_ = 3.38 ± 0.28 µg/mL) with no adverse effects at 0.006 µg/mL. Trypsin stability assays and molecular docking against serine proteases (trypsin and chymotrypsin) showed no significant off-target interactions. These findings suggest stigmasterol as a safe, selective, and effective natural tyrosinase inhibitor for potential use in skin-whitening formulation.

## Highlights

• Seaweed extracts were profiled using LC-MS and GC–MS techniques.
• *Gracilaria fisheri* showed the strongest anti-tyrosinase activity.
• Docking revealed stigmasterol as a strong tyrosinase inhibitor.
• Stigmasterol showed high safety, low toxicity, and good skin permeability.
• First report of seaweed-derived stigmasterol for skin whitening use.

## Graphical abstract

**Figure:**
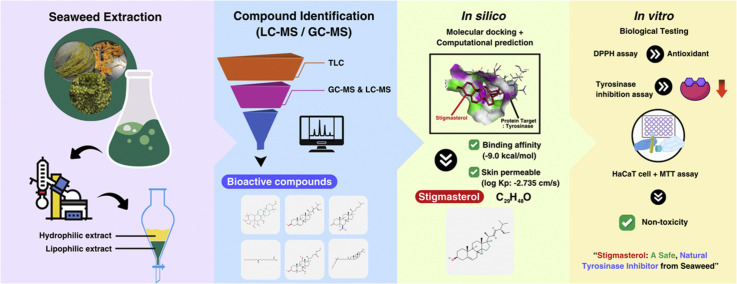

## 1 Introduction

The increasing global demand for natural and sustainable cosmetic ingredients has positioned marine bioresources, particularly seaweed, as promising sources of bioactive compounds. Seaweed is widely recognized for their diverse phytochemical profiles, which include polyphenols, polysaccharides, proteins, and other metabolites with significant applications in pharmaceuticals, nutraceuticals, and cosmetics [1,2]. This growing interest is driven by consumer preferences for environmentally responsible products and regulatory policies promoting sustainability, leading to the rapid integration of seaweed-derived extracts into the expanding cosmetics market.

Among the various bioactive compounds present in seaweed, phenolic compounds particularly phlorotannin have attracted significant attention due to their diverse dermatological benefits. These compounds exhibit potent antioxidants, anti-inflammatory, anti-aging, photoprotective, and skin-lightening properties, making them highly relevant for cosmetic applications [[3], [4], [5]]. Brown seaweeds, such as those from the *Sargassum* genus, are particularly rich in phenolic compounds and have demonstrated strong antioxidant and UV-protective activities [6]. Notably, *Sargassum siliquosum* and *Padina boergesenii* have exhibited substantial tyrosinase inhibitory activity, which is either comparable to or exceeds that of established skin-lightening agents such as kojic acid [7,8]. In addition to phenolic compounds, other bioactive constituents, such as mycosporine-like amino acids, fucoidan, and polysaccharides, contribute to the cosmetic efficacy of seaweed extracts by enhancing hydration, strengthening the skin barrier, and providing UV protection [3,6]. Furthermore, the natural thickening, gelling, and emulsifying properties of seaweed fractions support the formulation of innovative cosmetic products with desirable textures and functional performance [9].

Despite these promising attributes, challenges remain in utilizing seaweed-derived compounds in cosmetics. Variability in composition influenced by habitat, harvest time, environmental and seasonal factors, as well as potential degradation of bioactive compounds during extraction and purification, pose significant difficulties [1,[10], [11], [12]]. To address these challenges and maximize the potential of seaweed-derived bioactive compounds in cosmetics, precise identification and characterization of key compounds with specific skincare functions are essential. This targeted approach ensures consistency, efficacy, and safety in cosmetic formulations by isolating primary bioactive compounds and minimizing variability.

While previous studies have established the anti-tyrosinase activity of seaweed extracts, the specific compounds responsible for this effect remain largely unidentified, limiting the development of targeted therapies for hyperpigmentation. This study aims to address this knowledge gap by identifying and characterizing seaweed-derived bioactive compounds with potent anti-tyrosinase activity, thereby facilitating the development of sustainable and innovative skincare products. The research focuses on three native Thai seaweed species *Sargassum polycystum* (brown seaweed), *Caulerpa lentillifera* (green seaweed), and *Gracilaria fisheri* (red seaweed), which were intentionally selected based on their high abundance and availability along the Thai coastline, which enables sustainable harvesting. Additionally, these species represent diverse taxonomic groups, allowing for a broader exploration of chemical diversity across brown, green, and red macroalgae. While prior studies have indicated their general bioactivity, limited research has specifically examined their anti-tyrosinase potential, thus highlighting the novelty and relevance of this work. Methanol extraction will be employed to obtain crude extracts, which will then be fractionated into hydrophilic and lipophilic components for detailed chemical profiling using liquid chromatography-mass spectrometry (LC-MS) and gas chromatography-mass spectrometry (GC–MS). The extracts will be evaluated for their antioxidant and anti-tyrosinase activities to identify potential tyrosinase inhibitors. Extracts exhibiting strong inhibitory activity will undergo molecular docking and *in silico* toxicity assessments to predict their efficacy and safety. To further confirm dermal safety and target specificity, the lipophilic extracts and selected compounds will also be evaluated for potential interference with trypsin, a common proteolytic enzyme through enzyme stability assays. Additionally, molecular docking simulations will be conducted to predict selective binding affinities of these compounds toward trypsin and chymotrypsin. Finally, both the selected compounds and crude extracts will be validated through *in vitro* cytotoxicity testing using HaCaT human keratinocyte cells. This comprehensive methodological framework aims to establish a scientific basis for the sustainable utilization of seaweed-derived bioactive compounds in effective cosmetic formulations.

## 2 Materials and methods

### 2.1 Seaweed collection and preparation

Biomass of three seaweed species including *Sargassum polycystum* (Phaeophyta), *Caulerpa lentillifera* (Chlorophyta), and *Gracilaria fisheri* (Rhodophyta) was collected from various regions in Thailand. S*argassum polycystum* was obtained from Khanom Bay, Nakhon Si Thammarat Province, in southern Thailand. *Caulerpa lentillifera* was collected from Phetchaburi Province in eastern Thailand, while *Gracilaria fisheri* was sourced from Songkhla Province in southern Thailand. The collected samples were thoroughly washed with freshwater to remove excess salt and other impurities. The seaweed biomass was air-dried for two days and then completely dried using a hot-air oven (WTB Binder, Germany) set at 45–50 °C for a day and subsequently ground into fine particles using a mechanical grinder (BL42S166, Tefal, France). The resulting seaweed powder was stored in zip-lock bags to maintain its quality until further extraction procedures. In addition, seaweed was identified according to Abbot [13], Dawson [14], Egerod [15], Huisman [16], Lewmanomont and Ogawa [17] and confirmed by Assist. Prof. Dr. Bongkot Witchachucherd and Assist. Prof. Dr. Jantana Praiboon, Phycologist from Kasetsart University, Thailand.

### 2.2 Seaweed extraction and fractionation

A total of 1000 g of dried and ground seaweed samples were subjected to maceration in 3 L (1:3 w/v) of methanol for 7 days. The process was conducted in a dark cabinet at an ambient temperature to prevent the photodegradation of bioactive compounds. Following maceration, the methanolic extracts were filtered and concentrated using a rotary evaporator (Hei-VAP Precision, Heidolph, Germany) to obtain a viscous crude extract. The crude extract was partitioned between dichloromethane and distilled water (1:1 v/v), yielding lipophilic and hydrophilic fractions, respectively. The hydrophilic extracts were freeze-dried and stored in −20 °C, while the lipophilic extracts were evaporated to dryness and re-dissolved in ethanol at a concentration of 1 g of dried extract per 1 mL of ethanol and stored in amber glass bottles at a temperature range of 0–4 °C for subsequent experiments.

### 2.3 Seaweed extract analysis

#### 2.3.1 Thin-layer chromatography (TLC) analysis

For thin-layer chromatography (TLC) analysis, the hydrophilic and lipophilic extract was manually applied to aluminum TLC plates (20 × 20 cm, silica gel 60 F_254_, Merck, Darmstadt, Germany). After air-drying, the plates were placed in a saturated glass chamber using a mobile phase consisting of hexane and ethyl acetate (85:15 v/v). The TLC plates were sprayed with detecting reagents for screening major secondary metabolites including anisaldehyde-sulfuric acid reagent followed by heating on the TLC hotplate at 110 °C for 10 min for terpenoids detection, Dragendorff’s reagent for alkaloids detection and 10 % NaOH for coumarin detection. Compound bands were visualized through visual inspection and under UV light at wavelengths of 254 nm and 365 nm.

#### 2.3.2 Liquid chromatography mass spectrometry (LC-MS) analysis

The hydrophilic extract was investigated using a UPLC-DAD-Q-Orbitrap. The analysis was performed on a Vanquish UHPLC system (Thermo Fisher Scientific Inc.) equipped with a Thermo Scientific Vanquish- Binary Pump F, a Thermo Scientific Vanquish- Split Sampler FT, a Thermo Scientific Vanquish- Column Compartment H, and a Thermo Scientific Vanquish- Diode Array Detector FG. Mass spectrometry was done on a Thermo Scientific Orbitrap Exploris 120 system. The separation was done on a Kinetex 1.7 µm HILIC 100 Å LC column (50 × 2.1 mm). The mobile phase was constituted by 20 mM ammonium acetate (solvent A) and acetonitrile (solvent B) with linear gradient system as follows: 100 % B for 2 min, 100 % B to 0 % B in 8 min, 100 % A for 2 min. The flow rate was set at 0.5 mL min^-1^. Column temperature was controlled at 25 °C with still air. Diode array detection was set at a wavelength of 254 nm. The injection volume was 2 µL for all samples and standards.

For mass spectrometer, mass analysis was done in both positive and negative mode using internal mass calibration EASY-IC™. Ion source type was Heated-ESI. Spray voltage setting was static mode with positive ion 3500 V and negative ion 2500 V. Nitrogen gas mode was static with flow setting: sheath gas 60 Arb, Aux gas 15 Arb, and sweep gas 2 Arb. The Ion transfer tube temperature was 350 °C. Vaporizer temperature was 350 °C. Full scan mode range was 100–1000 m*/z* with resolution of 60,000 and RF Len 70 %. The ddMS^2^ mode was triggered with an intensity threshold of 5.0 × 10^5^. The MS^2^ parameters were isolation window: 1.5 m z^-1^, collision energy type: normalized, orbitrap resolution: 15,000, and scan range mode: automatic. The instrument was controlled and analyzed by Chromeleon™ Chromatography Data System software. Recorded chromatogram was visualized and analyzed using FreeStyle software. Compounds were identified or tentatively identified by their mass spectral data, compared with the National Institute of Standards and Technology (NIST) and mzCould database, or those reported in literature.

#### 2.3.3 Gas chromatography mass spectrometry (GC–MS) analysis

The lipophilic extract was dissolved in methanol (10:1 v/v), filtered through 0.45 µm membrane filter, and stored in glass vials. Gas chromatography-mass spectrometry (GC–MS) analysis was performed using an Agilent 8890 GC instrument (8890, Agilent Technologies, USA) equipped with an Agilent Technologies™ DB5MS column (Serial No 122–5532UI) consisting of cross-linked poly 5 % diphenyl and 95 % dimethylpolysiloxane (dimensions: 30 m x 0.25 mm, 0.25 µm film thickness). The system was operated in conjunction with MassHunter GC/MS software (7000D Triple Quadrupole, Agilent Technologies, USA). The initial oven temperature was programmed to be held at 100 °C for 3 min, followed by an increase to 160 °C at a rate of 10 °C per min, hold at 160 °C for 5 min. Subsequently, the temperature was raised to 280 °C at a rate of 5 °C per min, hold at 280 °C for 20 min. The transfer line, ion source temperatures were set at 280 °C and 230 °C, respectively. PAL3 autosampler machine injects liquid sample for 1 µL of the sample in Split mode, ratio 2:1. Flowrate 1.5 mL min^-1^ in constant control mode.

Mass spectral data were acquired at 70 eV in full-scan acquisition mode, covering the m z^-1^ range of 45–450. The GC–MS results provided. Total Ion Chromatogram (TIC) data along with match factor and reverse match factor scores. The analytical methodology adhered to ISO/IEC 17025 standards, ensuring rigorous quality control. Reproducibility was assessed based on retention times and mass spectra, with compound identification confirmed by comparison to the NIST Mass Spectral Search Program (Version 2.0) for the NIST/EPA/NIH Electron Ionization (EI) and Tandem Mass Spectral Library. The match factor score for library spectrum search results ranges from 700 to 800.

### 2.4 Antioxidant and anti-tyrosinase activities

#### 2.4.1 Antioxidant activity

The antioxidant activity of seaweed extracts was evaluated using the 2,2-diphenyl-1-picrylhydrazyl (DPPH) free radical scavenging assay, with modifications based on the method described by Zarei et al. [18]. A volume of 100 μL of the diluted seaweed extract was added to 100 μL of DPPH solution (0.15 mM) in a 96-well plate. The mixture was incubated in the dark at room temperature for 30 min, after which the absorbance was measured at 515 nm using microplate reader spectrophotometer (Sunrise, Tacan, Switzerland). Ascorbic acid was used as the standard reference antioxidant in this study. The antioxidant activity of the seaweed extracts was calculated using Eq. (1).

(1) $$\text{DPPH}\;\text{Inhibition}\;\left(\%\right)=[A_{\text{control}}-\left(A_{\text{sample}}-A_{\text{sampleblank}}\right)/A_{\text{control}}]x\;100$$

Where A_control_ represents the absorbance of DPPH solution, A_sample_ represents the absorbance of the sample solution and DPPH solution, and A_sampleblank_ represents the absorbance of sample solution without DPPH solution.

The DPPH inhibition percentage for the seaweed extracts was plotted, and the results were expressed as the IC_50_ value, defined as the concentration (μg mL^-1^) of extract required to achieve 50 % scavenging of the DPPH radical.

#### 2.4.2 Anti-tyrosinase activity

The tyrosinase inhibitory activity of seaweed extracts was evaluated using l-Dopa as a substrate, with modifications based on the methodology described by Liang et al. [19]. The reaction mixture (300 μL) consisted of 50 μL of mushroom tyrosinase enzyme (700 units/mL), 50 μL of the sample solution, and 100 μL of 0.1 M potassium phosphate buffer (pH 6.8), bringing the total volume to 200 μL. The mixture was incubated at room temperature for 10 min. Subsequently, 100 μL of 2.5 mM l-Dopa in 0.1 M potassium phosphate buffer (pH 6.8) was added to complete the reaction mixture. Following an additional incubation at room temperature for 20 min, the tyrosinase activity was measured by monitoring the formation of dopachrome at 475 nm using microplate reader spectrophotometer (Sunrise, Tacan, Switzerland). Kojic acid was used as a positive control in the assay. The anti-tyrosinase activity of the seaweed extracts was calculated using Eq. (2).

(2) $$\text{Tyrosinase}\;\text{Inhibition}\;\left(\%\right)=[A_{\text{control}}-\left(A_{\text{sample}}-A_{\text{sampleblank}}\right)/A_{\text{control}}]x\;100$$

Where A_control_ represents the absorbance of tyrosinase enzyme solution, A_sample_ represents the absorbance of the sample solution and tyrosinase enzyme solution, and A_sampleblank_ represents the absorbance of sample solution without tyrosinase enzyme solution.

The percentage inhibition of tyrosinase activity by the seaweed extracts was plotted, and the results were expressed as the IC_50_ value, which is defined as the concentration (µg mL^-1^) of the extract required to achieve 50 % inhibition of tyrosinase activity.

### 2.5 Cell toxicity test

The immortalized human keratinocyte HaCaT cell line provided by Professor Kesara Na-Bangchang was used as a cell model in the cytotoxicity test. HaCaT cells were maintained in Dulbecco's Modified Eagle Medium (DMEM: Gibco Co. Ltd, Grand Island, NY, USA) in a 5 % CO_2_ humidified incubator at 37 °C. HaCaT cells were seeded in a 96-well plate at a density of 1.2 × 10^4^ cells well^-1^ and incubated overnight at 37 °C in a cell culture incubator with humidified atmosphere of 5 % CO_2_. The cells were exposed to tested compounds for 24 h and cell viability was examined using MTT assay (3, [4, 5-dimethylthiazol-2-yl]−2, 5-diphenyltetrazolium bromide (Invitrogen, CA, USA). Briefly, 20 µL of 5 mg mL^-1^ of MTT in PBS was added to each well of the 96-well plate and the plate was incubated for 3 h in the cell culture incubator. Then 100 µL of DMSO (Chem Cruz, CA, USA) was added in each well to dissolve formazan crystals and optical density (OD) was measured by SPECTROstar ^Nano^ microplate reader (BMG LABTECH,Ortenberg, Germany) at a wavelength of 590 nm. Percentage of cell viability was calculated as Eq. (3).

(3) $$\text{Cell}\;\text{viability}\left(\%\right)=\left[\left(OD_{\text{treatedcell}}--OD_{\text{negativecontrol}}\right)/\left(OD_{\text{control}}--OD_{\text{negativecontrol}}\right)\right]\times 100$$

Where OD_treatedcell_ represents the absorbance of formazan solution from treated cells, OD_control_ represents the absorbance of formazan solution from untreated cells, and OD_negativecontrol_ represents the absorbance of DMSO solution.

### 2.6 Proteinase stability assay

To evaluate the proteolytic stability of bioactive compounds, a small-scale enzymatic degradation assay was conducted using trypsin, a common serine protease. Crude lipophilic extracts from three seaweeds and stigmasterol were prepared at twice their IC_50_ concentrations in ethanol. Each sample was tested under three conditions including 800 µL of extract with 200 µL of 0.25 % trypsin in PBS to assess enzymatic degradation, 800 µL of extract with 200 µL of phosphate-buffered saline (PBS, pH 7.4) serving as the positive control, and 800 µL of PBS with 200 µL of 0.25 % trypsin as the negative control. The mixtures were incubated at 25 °C for 25 min, after which enzymatic activity was terminated by the addition of 10 µL of a protease inhibitor. Samples were then diluted to their original IC_50_ concentrations and subjected to a standard anti-tyrosinase assay to determine post-treatment activity.

### 2.7 *In silico* analysis

#### 2.7.1 Identification of bioactive compound performance on anti-tyrosinase activity using computational approaches

##### 2.7.1.1 Protein preparation

The three-dimensional structures of the proteins were retrieved from the RCSB Protein Data Bank (https://www.rcsb.org) in PDB format. The study utilized the X-ray crystal structure of mushroom tyrosinase (PDB ID: 2Y9X) [20,21], which consists of 391 amino acids derived from *Agaricus bisporus*, complexed with the inhibitor tropolone, and resolved at 2.78 Å. Additionally, the crystal structure of human tyrosinase related protein 1 (PDB ID: 5M8L) [22], containing 446 amino acids from *Homo sapiens*, was used with a resolution of 2.35 Å. Before docking studies, ligand molecules, non-interacting ions, and water molecules were removed from the protein structures using BIOVIA Discovery Studio Client 2021. Subsequently, polar hydrogens were added, and Kollman charges were assigned to the receptors. The prepared receptor structures were then saved in PDBQT format using AutoDock Tools (ADT) version 1.5.7. Binding sites of the proteins were predicted using the PrankWeb binding site prediction tool to facilitate further molecular docking analyses.

##### 2.7.1.2 Ligand preparation

A library of 56 bioactive compounds from *G. fisheri*, including tropolone (PubChem CID: 10789) and kojic acid (PubChem CID: 3840) [23], known for their anti-tyrosinase activity, was retrieved from the PubChem database (https://pubchem.ncbi.nlm.nih.gov/) as SDF structural files. The three-dimensional structures of all ligand molecules were generated and converted to PDB format using the Open Babel module [24]. The ligand structures were optimized to achieve their lowest energy conformations using Avogadro [25]. Chemical structures were further refined using the Clean Geometry tool in BIOVIA Discovery Studio Client 2021. Polar hydrogens were added, and Kollman charges were assigned to the ligands. Rotatable bond torsions were defined for each ligand, and Gasteiger charges were calculated for the energy-minimized conformations using AutoDock Tools (ADT) version 1.5.7 [26]. Finally, the prepared ligand structures were saved in PDBQT file format for subsequent docking studies.

##### 2.7.1.3 Molecular docking

AutoDock Vina v.1.2.0 was employed for molecular docking simulations via the Linux command line interface. During the docking process, the target proteins were treated as rigid, while the ligands were modeled as flexible to allow conformational adjustments. For the mushroom protein, the grid box was configured with dimensions of 72 × 60 × 70 Å (X, Y, and Z axes, respectively) and centered at coordinates −7.620, −24.771, and −38.576 (center X, Y, and Z, respectively), corresponding to the binding site on the target protein [23]. For human tyrosinase, the grid box was defined with dimensions of 40 × 40 × 40 Å and centered at coordinates 43.1758, 122.8245, and 183.5534. The optimal docking pose for each protein-ligand complex was determined by selecting the conformation with the lowest binding energy (kcal/mol). Ligands exhibiting binding energies equal to or lower than −8.0 kcal mol^-1^ were shortlisted for further analysis. Visualization and detailed inspection of the best-docked protein-ligand complexes were performed using BIOVIA Discovery Studio Client 2021.

#### 2.7.2 Molecular docking of seaweed-derived compounds against proteolytic enzymes

Molecular docking simulations were performed to assess the binding affinities of eight lead compounds, retrieved from the PubChem database (https://pubchem.ncbi.nlm.nih.gov/), against two serine proteases including trypsin (PDB ID: 2ZQ1) and alpha-chymotrypsin (PDB ID: 4Q2K). The protein crystal structures were obtained from the RCSB Protein Data Bank (https://www.rcsb.org) in PDB format. A consistent grid box size of 20 × 20 × 20 Å was applied across all docking simulations to maintain uniformity in the ligand search space. The grid box centers were determined based on the coordinates of the co-crystallized ligands within each protein, −26.690, 1.232, and 14.565 for trypsin and 61.757, 1.189, and 28.006 for alpha-chymotrypsin. Docking calculations were conducted using AutoDock software, and the binding affinities for each compound and protein interaction were recorded in kcal/mol.

#### 2.7.3 Skin absorption and toxicity prediction

The selected molecules were evaluated for skin absorption and toxicity using the pkCSM server (https://biosig.lab.uq.edu.au/pkcsm/) [27]. This computational platform provides pharmacokinetic predictions critical for the development of skincare products, with a specific focus on skin absorption properties. Additionally, toxicity assessments were conducted to evaluate potential risks, including skin sensitization, carcinogenicity, AMES mutagenicity, and hepatotoxicity.

### 2.8 Data analysis

Chemical composition similarity based on present and absent data between seaweed species was computed using Sørenson’s coefficient similarity index (S) as Eq. (4).

(4) $$\begin{array}{c} S=2a/\left(2a+b+c\right) \end{array}$$

Where a is the number of compounds present in both species, b is the number of compounds present in species 1 and c is the number of compounds present in species 2.

The experimental data were reported as average ± standard deviation with three independent experiments. To compare the treatments of the antioxidant and anti-tyrosinase activities and cell cytotoxicity experiments, a one-way analysis of variance (one-way ANOVA) was performed at *p* < 0.05 level, and Tukey’s HSD post hoc analysis was used for multiple comparisons of each treatment (*p* ≤ 0.05). All data analysis was computed using IBM SPSS statistics 23. Statistical analysis of cell viability compared to untreated control cells was performed using one-way ANOVA followed by Dunnett’s multiple comparison post hoc test using Prism version 10.2.

## 3 Results

### 3.1 Yield percentage of seaweed extracts

The yield percentages of hydrophilic and lipophilic crude extracts obtained from three seaweed species using methanol as the solvent showed variability in the extract yields among the species (Table 1). *G. fisheri* showed the significantly highest hydrophilic yield, producing 17.85 ± 0.24 g of crude extract, which corresponds to a yield of 1.85 % (*p* ≤ 0.05). In contrast, *C. lentillifera* demonstrated the significantly highest lipophilic yield, producing 24.94 ± 0.19 g of crude extract, equivalent to a yield of 2.49 ± 0.03 % (*p* ≤ 0.05).

**Table 1:** Yield percentages of lipophilic crude extracts from three seaweed species.

**Seaweed Species**	**Hydrophilic extract** **(g)**	**Yield** **( %)**	**Lipophilic Extract (g)**	**Yield** **( %)**
*S. polycystum*	11.56^c^ ± 0.46	1.16^c^ ± 0.05	10.48^c^ ± 0.04	1.05^c^ ± 0.01
*C. lentillifera*	15.46^b^ ± 0.56	1.55^b^ ± 0.06	24.94^a^ ± 0.19	2.49^a^ ± 0.02
*G. fisheri*	17.85^a^ ± 0.24	1.79^a^ ± 0.02	13.30^b^ ± 0.26	1.33^b^ ± 0.03

Data are presented as mean ± standard deviation (*n* = 3). Superscript letters (a, b, c) within each column indicate significant differences between seaweed species based on one-way ANOVA followed by Tukey's HSD post hoc test (*p* < 0.05).

### 3.2 Seaweed extract analysis by TLC, LC-MS and GC–MS

The TLC plate analysis revealed that the lipophilic extracts of *S. polycystum, C. lentillifera*, and *G. fisheri* contained distinct chemical components, including terpenoids, alkaloids, and coumarins. The lipophilic extracts exhibited consistent Rf values of 0.26, 0.58, and 0.68 for terpenoids, alkaloids, and coumarins, respectively, across all three species. In contrast, the hydrophilic extracts primarily contained terpenoids, with Rf values ranging from 0.50 to 0.57. Additionally, alkaloids were detected in the hydrophilic extract of *S. polycystum*, with an Rf value of 0.24. These findings demonstrate the presence of bioactive compounds in the seaweed extracts, emphasizing their potential for various biotechnological applications (Supplementary Fig. 1 and Supplementary Table 1).

The comprehensive chemical profiling of seaweed extracts using LC-MS and GC–MS analyses revealed a diverse range of bioactive compounds in both hydrophilic and lipophilic fractions. The LC-MS analysis of hydrophilic extracts identified key bioactive compounds, including C16 phytosphingosine, thiamine, acetylcholine, and tropolone A, as well as more complex molecules such as steroidal derivatives and N2-[4-({[3-(Cyclohexylamino)propyl]amino}methyl)benzyl]−6-(1-piperazinyl)-2,4-pyrimidinediamine. These compounds displayed varying distributions, with some shared among the three seaweed species, while others were unique to specific species. The retention times ranged from 0.35 to 7.55 min, with molecular weights and corresponding *m/z* ratios confirming their identities (Supplementary Table 2).

**Fig. 1:**
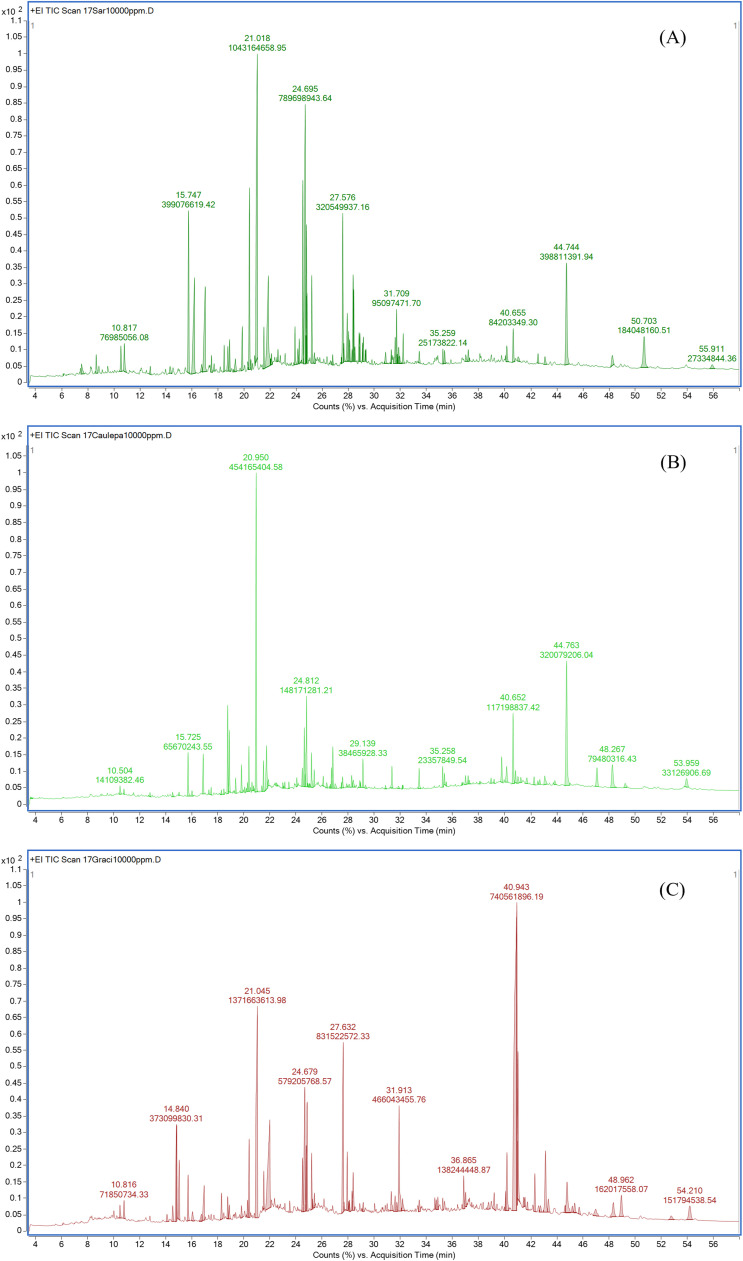
Total ion chromatogram (TIC) profile of the lipophilic extracts from (A) *Sargassum polycystum*, (B) *Caulerpa lentillifera* and (C) *Gracilaria fisheri* analyzed by GC–MS. The chromatograms display distinct chemical profiles for each species, with major peaks indicating the presence of bioactive lipophilic compounds.

Meanwhile, the GC–MS chromatographic profiles of lipophilic extracts from the three seaweed species confirmed the presence of numerous compounds within the methanol extracts. A total of 112 chemical components were identified, with nine compounds commonly present across the three species, including Propanoic acid, 2-(3-acetoxy-4,4,14-trimethylandrost-8-en-17-yl)-, Tridecanoic acid, 12-methyl-, methyl ester (Methyl 12-methyltridecanoate), 6-Hydroxy-4,4,7a-trimethyl-5,6,7,7a-tetrahydrobenzofuran-2(4H)-one, Hexadecanoic acid, methyl ester (Methyl palmitate), n-Hexadecanoic acid (Palmitic acid), Ethanol, 2-(9-octadecenyloxy)-, (Z)- (Emulphor), (Z)- (emulphor), Ethyl iso-allocholate (Ethyl cholate), 1-Heptatriacontanol, and Hexadecanoic acid, 1-(hydroxymethyl)-1,2-ethanediyl ester (1,2-Dipalmitoyl-rac-glycerol). These findings, detailed in Supplementary Table 3 and presented in Fig. 1. A comparative analysis of the chemical compositions revealed that *S. polycystum* shared a 42.52 % compound similarity to *C. lentillifera* and a 38.94 % similarity to *G. fisheri*. Additionally, *C. lentillifera* exhibits a 40.34 % similarity to *G. fisheri*. These results indicate that each of the three seaweeds is a rich source of bioactive compounds, with intergroup dissimilarity exceeding 61.1 %.

### 3.3 Antioxidant and anti-tyrosinase activities

The DPPH radical scavenging activity of crude extracts from three seaweed species was assessed using ascorbic acid as the standard. Ascorbic acid showed the significant highest activity. Among the seaweed extracts, *S. polycystum* showed the strongest activity, followed by *G. fisheri* and *C. lentillifera*, respectively (Table 2). These results highlight *S. polycystum* as the most effective antioxidant among the tested species. In addition, the anti-tyrosinase activity of crude lipophilic extracts from *S. polycystum, C. lentillifera*, and *G. fisheri* was assessed, with kojic acid as the standard. The IC_50_ values showed that *G. fisheri* presented the highest activity, followed by *C. lentillifera*, while *S. polycystum* exhibited the lowest activity. Notably, the crude hydrophilic and lipophilic extract of *G. fisheri* demonstrated the highest anti-tyrosinase activity among the three seaweed species studied. To identify the significant chemical compounds in the *G. fisheri* extract contributing to this inhibitory effect on tyrosinase, an enzyme involved in melanin production, a computational screening approach was employed.

**Table 2:** The DPPH radical scavenging and anti-tyrosinase activities of hydrophilic and lipophilic extracts from *S. polycystum, C. lentillifera* and *G. fisheri* and stigmasterol.

**Sample**	**Hydrophilic extract** **IC_50_ (µg mL^-1^)**	**Lipophilic extract** **IC_50_ (µg mL^-1^)**
DPPH radical scavenging activity		
Ascorbic acid	3.03^a^ ± 0.03	3.03^a^ ± 0.03
*S. polycystum*	10,279.98^b^ ± 75.25	911.54^b^ ± 5.76
*C. lentillifera*	15,621.75^c^ ± 188.41	2701.54^d^ ± 59.64
*G. fisheri*	10,420.25^b^ ± 119.18	1084.67^c^ ± 71.25
Anti-tyrosinase activity		
Kojic acid	9.78^b^ ± 0.17	9.78^c^ ± 0.17
*S. polycystum*	0.19^a^ ± 0.01	4.32^b^ ± 0.50
*C. lentillifera*	0.33^a^ ± 0.02	0.21^a^ ± 0.07
*G. fisheri*	0.21^a^ ± 0.02	0.15^a^ ± 0.05
Stigmasterol	–	0.16^a^ ± 0.01

Data are presented as mean ± standard deviation (*n* = 3). Superscript letters (a, b, c) within each column indicate significant differences between seaweed species based on one-way ANOVA followed by Tukey’s HSD post hoc test (*p* < 0.05).

### 3.3 *In silico* analysis

#### 3.3.1 Binding affinities of seaweed-derived compounds with mushroom and human tyrosinase

**Fig. 2:**
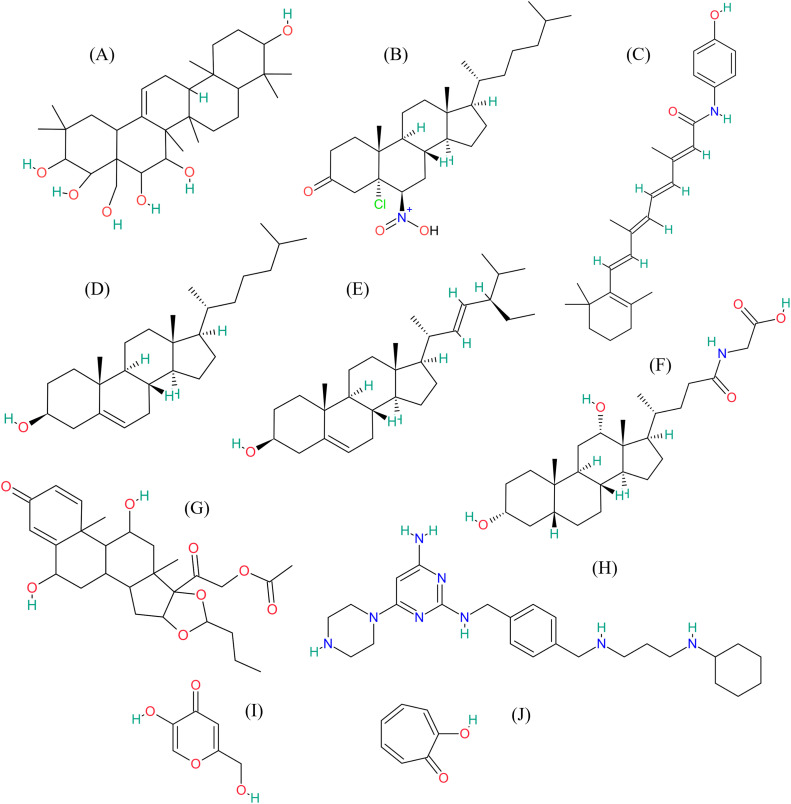
Two-dimensional chemical structures of the top eight lead compounds in *G. fisheri* extract after molecular docking analysis. (A) Olean-12-ene-3,15,16,21,22,28-hexol, (3β,15α,16α,21β,22α)-; (B) 5-Chloro-6beta-nitro-5alpha-cholestan-3-one; (C) Fenretinide; (D) Cholesterol; (E) Stigmasterol; (F) Glycodeoxycholic acid; (G) (22S)-21-Acetoxy-6α,11β-dihydroxy-16α,17α-propylmethylenedioxypregna-1,4-diene-3,20‑dione; (H) N∼2∼-[4-({[3-(Cyclohexylamino)propyl]amino}methyl)benzyl]−6-(1-piperazinyl)-2,4-pyrimidinediamine; (I) Kojic acid, and (J) Tropolone.

Among the three seaweed species tested, *G. fisheri* exhibited the highest anti-tyrosinase activity. Consequently, bioactive compounds derived from this red seaweed were selected to construct a data library for computational analysis. Molecular docking studies were performed on 56 bioactive compounds from *G. fisheri* extract, along with two control inhibitors, kojic acid and tropolone, targeting both mushroom and human tyrosinase. For mushroom tyrosinase, the binding affinities of the tested compounds ranged from −4.5 kcal mol^-1^ to −9.8 kcal mol^-1^, with Olean-12-ene-3,15,16,21,22,28-hexol (3β,15α,16α,21β,22α) demonstrating the strongest binding affinity at −9.8 kcal mol^-1^, followed by Stigmasterol at −9.6 kcal mol^-1^. For human tyrosinase, binding affinities ranged from −5.7 kcal mol^-1^ to −10.2 kcal mol^-1^, with Fenretinide and N∼2∼-[4-({[3-(Cyclohexylamino)propyl]amino}methyl)benzyl]−6-(1-piperazinyl)-2,4-pyrimidinediamine displaying the highest affinity at −10.2 kcal mol^-1^, while both Stigmasterol and Olean-12-ene-3,15,16,21,22,28-hexol (3β,15α,16α,21β,22α) exhibited binding energies of −8.6 kcal mol^-1^. Overall, eight compounds were identified with binding affinity of −8.0 kcal/mol or lower for both tyrosinase targets, as detailed in Table 3 and Fig.2. Among the 56 compounds analyzed, these eight compounds were selected as the most promising lead compounds based on their molecular docking results and will be further analyzed for skin permeability and toxicity using computational approaches.

**Table 3:** Binding affinities (kcal mol^-1^) of the top eight lead compounds in *G. fisheri* extract with mushroom and human tyrosinase.

**Compound name**	**Formula**	**Mushroom** **tyrosinase** **(2Y9X)** **Binding energy (kcal mol^-1^)**	**Human** **tyrosinase (5M8L)** **Binding energy (kcal mol^-1^)**
Olean-12-ene-3,15,16,21,22,28-hexol, (3β,15α,16α,21β,22α)-	C_30_H_50_O_6_	−9.0	−8.1
5-Chloro-6beta-nitro-5alpha-cholestan-3-one	C_27_H_44_ClNO_3_	−8.6	−10.2
Fenretinide	C_26_H_33_NO_2_	−9.0	−8.1
Cholesterol	C_27_H_46_O	−8.6	−10.2
Stigmasterol	C_29_H_48_O	−9.0	−8.1
Glycodeoxycholic acid	C_26_H_43_NO_5_	−8.6	−10.2
(22S)-21-Acetoxy-6α,11β-dihydroxy-16α,17α-propylmethylenedioxypregna-1,4-diene-3,20-dione	C_27_H_36_O_8_	−9.0	−8.1
N∼2∼-[4-({[3-(Cyclohexylamino)propyl]amino}methyl)benzyl]−6-(1-piperazinyl)-2,4-pyrimidinediamine	C_25_H_40_N_8_	−8.6	−10.2
Control Inhibitors			
Kojic acid	C_6_H_6_O_4_	−5.5	−5.7
Tropolone	C_7_H_6_O_2_	−5.9	−5.8

#### 3.3.2 Molecular interactions and surface analysis

**Fig. 3:**
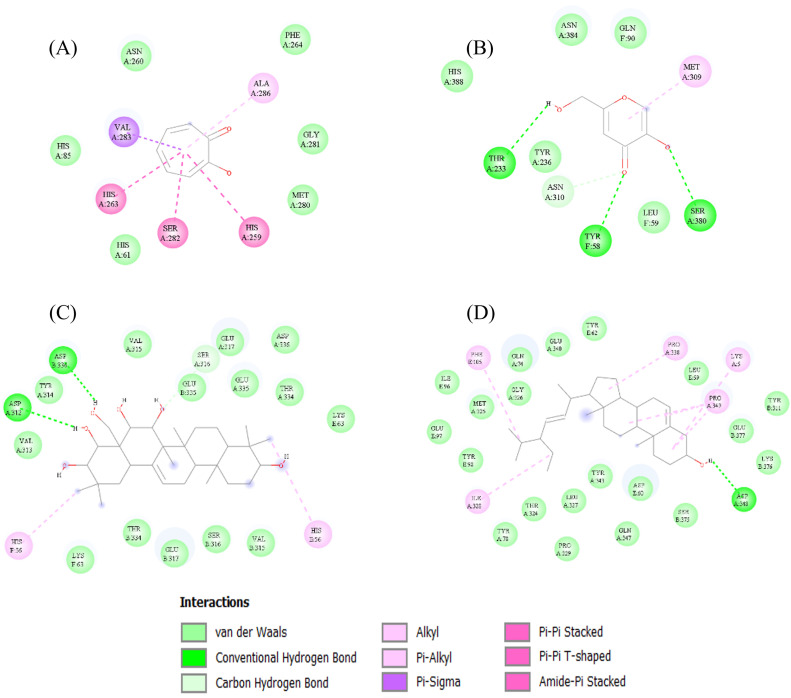
Two-dimensional molecular interaction diagrams showing the interacting amino acid residues of mushroom tyrosinase (PDB ID: 2Y9X) with control inhibitors and potential bioactive compounds from *G. fisheri* extract. (A) Tropolone (PubChem CID: 10789), (B) Kojic acid (PubChem CID: 3840), (C) Olean-12-ene-3,15,16,21,22,28-hexol, (3β,15α,16α,21β,22α) and (D) Stigmasterol.

**Fig. 4:**
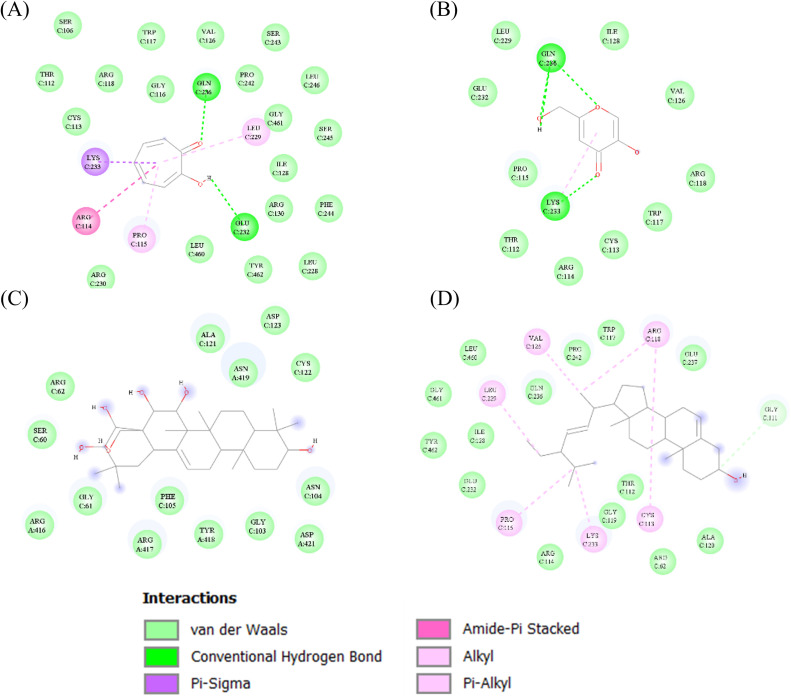
Two-dimensional molecular interaction diagrams showing the interacting amino acid residues of human tyrosinase (PDB ID: 5M8L) with control inhibitors and potential bioactive compounds from *G. fisheri* extract. (A) Tropolone (PubChem CID: 10789), (B) Kojic acid (PubChem CID: 3840), (C) Olean-12-ene-3,15,16,21,22,28-hexol, (3β,15α,16α,21β,22α) and (D) Stigmasterol.

Molecular interactions between the selected compounds and tyrosinase were analyzed to examine hydrogen bonding, hydrophobic interactions, and Pi interactions, as summarized in Table 4. For mushroom tyrosinase, Olean-12-ene-3,15,16,21,22,28-hexol formed hydrogen bonds with ASP312 and ASP336, hydrophobic interactions with several residues, and Pi interactions with HIS56. Stigmasterol exhibited similar interactions, forming hydrogen bonds with ASP348, hydrophobic and Pi interactions with multiple residues. In contrast, tropolone displayed only hydrophobic and Pi interactions, while kojic acid formed hydrogen bonds, hydrophobic interactions, and Pi interactions with different residues. For human tyrosinase, Olean-12-ene-3,15,16,21,22,28-hexol exhibited only hydrophobic interactions, whereas stigmasterol formed both hydrophobic and Pi interactions. Tropolone and kojic acid exhibited similar patterns, involving hydrogen bonding, hydrophobic, and Pi interactions across multiple residues. These molecular interactions are illustrated in Fig. 3, Fig. 4.

**Table 4:** Interacting with amino acid residues for the top eight lead compounds of seaweed metabolites.

**Protein Target**	**PDBID**	**Ligand Name**	**Binding Affinity Score**	**Interactions**
Mushroom tyrosinase	2Y9X	Tropolone	−5.9	**Hydrogen Bonds**: **Hydrophobic Interactions**: HIS61, HIS85, ASN260, PHE264, MET280, GLY281 **Pi interactions**: HIS259, HIS263, SER282, VAL283, ALA286
Mushroom tyrosinase	2Y9X	Kojic acid	−5.5	**Hydrogen Bonds:** TYR58, THR233, SER380 **Hydrophobic Interactions:** LEU59, GLN90, TYR236, ASN310, ASN384, HIS388 **Pi interactions:** MET309
Mushroom tyrosinase	2Y9X	Olean-12-ene-3,15,16,21,22,28-hexol, (3β,15α,16α,21β,22α)-	−9.8	**Hydrogen Bonds:** ASP312, ASP336 **Hydrophobic Interactions:** LYS63, VAL313, VAL315, SER316. GLU317, THR334, GLU335,ASP336 **Pi interactions:** HIS56
Mushroom tyrosinase	2Y9X	Stigmasterol	−9.6	**Hydrogen Bonds:** ASP348 **Hydrophobic Interactions:** LEU59, ASP60, TYR62, GLN74, TYR78, ILE96, GLU97, TYR98, TYR311, THR324, MET325, GLY326, LEU327, PRO329, GLU340, TYR343, GLN347, SER375, LYS376, GLU377 **Pi interactions:** LYS5, PHE105, ILE328, PRO338, PRO349
Human tyrosinase	5M8L	Tropolone	−5.8	**Hydrogen Bonds:** GLN236, GLU232 **Hydrophobic Interactions:** SER106, THR112, CYS113, GLY116, TRP117, ARG118, VAL126, ILE128, ARG130, LEU228, ARG230, PRO242, SER243, PHE244, SER245, LEU246, LEU460, GLY461, TYR462 **Pi interactions:** ARG114, PRO115, LEU229, LYS233
Human tyrosinase	5M8L	Kojic acid	−5.7	**Hydrogen Bonds:** LYS233, GLN236 **Hydrophobic Interactions:** THR112, CYS113, ARG114, PRO115, TRP117, ARG118, VAL126, ILE128, LEU229, GLU232 **Pi interactions:** LYS233
Human tyrosinase	5M8L	Olean-12-ene-3,15,16,21,22,28-hexol, (3β,15α,16α,21β,22α)-	−8.6	**Hydrogen Bonds:** **Hydrophobic Interactions:** SER60, GLY61, ARG62, GLY103, ASN104, PHE105, ALA121, CYS122, ASP123, ARG416, ARG417, TYR418, ASN419, ASP421 **Pi interactions:**
Human tyrosinase	5M8L	Stigmasterol	−8.6	**Hydrogen Bonds:** **Hydrophobic Interactions:** ARG62, GLY111, THR112, ARG114, TRP117, GLY119, ALA120, ILE128, GLU232, GLN236, GLU237, PRO242, LEU460, GLY461, TYR462 **Pi interactions:** CYS113, PRO115, ARG118, VAL126, LEU229, LYS233

Molecular surface analysis further elucidated the spatial arrangement of the compounds within the protein-binding pocket, highlighting key interactions that may contribute to binding stability. The analysis revealed distinct regions of electron donor and acceptor properties, represented in purple and green, respectively (Supplementary Fig. 2 and Supplementary Fig.3). These insights into the molecular surface provide a clearer understanding of how the compounds interact with the binding pocket and contribute to their overall binding affinity and stability.

#### 3.3.4 Skin absorption and toxicity assessment

The pkCSM server analysis indicated favorable skin permeability for all selected compounds, with log Kp values ranging from −3.157 cm s^-1^ for kojic acid to −2.735 cm s^-1^ for Glycodeoxycholic acid. All values exceeded the established threshold of −7.0 cm s^-1^, suggesting effective skin absorption potential [23]. Among the tested compounds, Glycodeoxycholic acid exhibited the highest permeability (log Kp = −2.735 cm s^-1^), followed by N∼2∼-[4-({[3-(Cyclohexylamino) propyl] amino} methyl) benzyl]−6-(1-piperazinyl)-2,4-pyrimidinediamine (log Kp = −2.743 cm s^-1^), 5-Chloro-6β-nitro-5α-cholestan-3-one (log Kp = −2.748 cm s^-1^), and Stigmasterol (log Kp = −2.783 cm s^-1^), with kojic acid demonstrating the lowest permeability (log Kp = −3.157 cm s^-1^) (Table 5). Toxicity predictions revealed that none of the compounds, including kojic acid, showed AMES toxicity or skin sensitization, except for tropolone, which was predicted to have skin sensitization potential. Hepatotoxicity was identified in three compounds including Fenretinide, Glycodeoxycholic acid and N∼2∼-[4-({[3-(Cyclohexylamino) propyl] amino} methyl) benzyl]−6-(1-piperazinyl)-2,4-pyrimidinediamine, while the remaining compounds were predicted to be non-hepatotoxic. Among the analyzed compounds, Stigmasterol and 5-Chloro-6β-nitro-5α-cholestan-3-one demonstrated a favorable balance of high skin permeability, absence of AMES toxicity, and lack of skin sensitization or hepatotoxicity. These characteristics, coupled with their anti-tyrosinase activity, highlight their potential as effective components in cosmetic formulations targeting hyperpigmentation and skin whitening. These findings suggest their suitability for use in cosmetic applications and their promise as safe and effective anti-tyrosinase agents.

**Table 5:** Predicted skin absorption and toxicity assessment of top lead compounds by pkCSM Server.

**Compound name**	**Skin** **Permeability (log Kp≥−7.0cm s^-1^)**	**AMES toxicity**	**Hepatotoxicity**	**Skin** **sensitization**
Olean-12-ene-3,15,16,21,22,28-hexol, (3β,15α,16α,21β,22α)-	−2.855	No	No	No
5-Chloro-6beta-nitro-5alpha-cholestan-3-one	−2.748	No	No	No
Fenretinide	−2.922	No	Yes	No
Cholesterol	−2.841	No	No	No
Stigmasterol	−2.783	No	No	No
Glycodeoxycholic acid	−2.735	No	Yes	No
(22S)-21-Acetoxy-6α,11β-dihydroxy-16α,17α-propylmethylenedioxypregna-1,4-diene-3,20-dione	−3.082	Yes	No	No
N∼2∼-[4-({[3-(Cyclohexylamino) propyl] amino} methyl) benzyl]−6-(1-piperazinyl)-2,4-pyrimidinediamine	−2.743	No	Yes	No
Control Inhibitors				
Kojic acid	−3.157	No	No	No
Tropolone	−3.078	No	No	Yes

Stigmasterol, a plant-derived sterol, is generally more affordable due to the abundance of raw materials and the efficiency of large-scale production processes [28]. In contrast, 5-Chloro-6β-nitro-5α-cholestan-3-one must be synthesized from cholesterol through chemical modifications, resulting in higher production costs due to the complexity of its synthetic process [29]. Stigmasterol is widely utilized in the pharmaceutical and health supplement industries because of its natural origin and beneficial properties, whereas 5-Chloro-6β-nitro-5α-cholestan-3-one is primarily used as a research compound due to its limited availability and specialized applications. Based on these factors, stigmasterol was selected for further investigation, including *in vitro* cell toxicity analysis.

### 3.4 Cell cytotoxicity

**Fig. 5:**
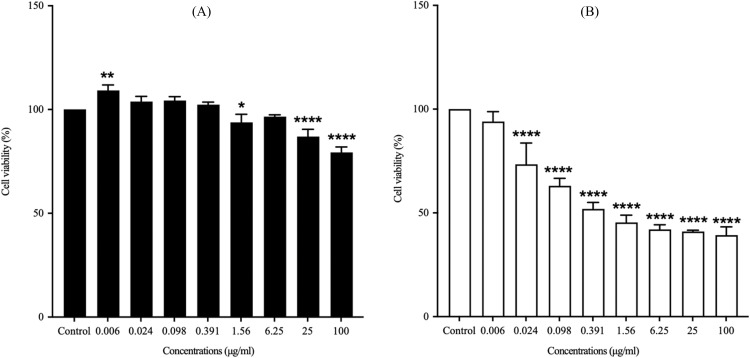
Effect of (A) crude lipophilic extract of *G. fisheri* and (B) stigmasterol on the viability of HaCaT keratinocyte cells after 24 h exposure, as measured by the MTT assay. Results are expressed as mean ± SD (*n* = 3). Statistical differences in cell viability compared to untreated control cells were analyzed using one-way ANOVA followed by Dunnett’s multiple comparison post hoc test. Significance levels are indicated as follows: **** *P* < 0.0001, ** *P* < 0.01, and * *P* < 0.05 compared to control.

The cytotoxicity evaluation of stigmasterol and the crude lipophilic extract of *G. fisheri* revealed distinct bioactivity profiles. At lower concentrations (≤25 μg mL^-1^), the crude extract maintained cell viability above 70 %, indicating limited toxicity within this range. Furthermore, the extract exhibited no harmful effects on skin cells at concentrations below 0.391 μg mL^-1^, where cell viability remained unaffected (Fig. 5A). However, at higher concentrations (25.0 μg mL^-1^), a significant reduction in cell viability was observed, suggesting potential cytotoxic effects at elevated levels. The IC_50_ value of the crude extract was determined to be 161.0±23.13 μg mL^-1^, indicating relatively low cytotoxicity compared to stigmasterol. In contrast, stigmasterol demonstrated significantly higher cytotoxicity, with an IC_50_ value of 3.38 ± 0.28 μg mL^-1^. Stigmasterol substantially reduced cell viability even at low concentrations, reflecting its potent bioactivity. Nonetheless, it was non-toxic to skin cells at concentrations as low as 0.006 μg mL^-1^ (Fig. 5B), suggesting that it may be safely applied at very low concentrations.

### 3.5 Trypsin stability of seaweed extracts and stigmasterol

The results indicated that the anti-tyrosinase activity of all seaweed extracts and stigmasterol remained largely unaffected following trypsin exposure. The percentage inhibition in the trypsin-treated samples closely matched those in the untreated positive controls, suggesting no significant loss in activity. Specifically, inhibition values for all extracts and stigmasterol ranged from 49.17±2.39 % to 50.79±1.18 % after trypsin treatment, comparable to their pre-treatment values (Table 6). Conversely, the negative control containing trypsin alone consistently showed lower inhibition levels, confirming that trypsin does not possess intrinsic tyrosinase-inhibitory properties. These findings suggest that the tested seaweed-derived compounds and stigmasterol exhibit resilience to proteolytic degradation by trypsin, supporting their potential stability and functionality in enzyme-rich biological environments such as the skin.

**Table 6:** Anti-tyrosinase activity of seaweed extracts and stigmasterol before and after trypsin treatment. Enzyme-treated sample (800 µL of extract + 200 µL of 0.25 % trypsin in PBS), Positive control (800 µL of extract + 200 µL PBS) and Negative control (800 µL of PBS + 200 µL of 0.25 % trypsin).

Sample	Enzyme-treated sample ( % inhibiton)	Positive control ( % inhibiton)	Negative control ( % inhibiton)
*S. polycystum*	50.00^a^ ± 1.18	50.39^a^ ± 1.18	31.50^b^ ± 0.68
*C. lentillifera*	49.17^a^ ± 2.39	50.39^a^ ± 1.36	31.89^b^ ± 1.80
*G. fisheri*	50.79^a^ ± 1.18	51.18^a^ ± 1.18	31.10^b^ ± 1.18
Stigmasterol	49.21^a^ ± 1.18	50.39^a^ ± 2.36	31.10^b^ ± 1.80

Data are presented as mean ± standard deviation (*n* = 3). Superscript letters (a, b, c) within each row indicate significant differences between seaweed species based on one-way ANOVA followed by Tukey’s HSD post hoc test (*p* < 0.05).

### 3.6 Binding affinities of seaweed-derived compounds with serine proteases

Molecular docking analyses revealed that the top eight seaweed-derived compounds exhibited moderate binding affinities toward the serine proteases trypsin and alpha-chymotrypsin, with binding affinities generally lower than those of their respective control inhibitors (Table 7). This suggests that these enzymes are unlikely to be primary targets of the eight compounds. When compared to their binding affinities against tyrosinase (Table 3), the compounds demonstrated a stronger preference for tyrosinase, implying a degree of target selectivity. Stigmasterol displayed weaker binding affinity with trypsin (−7.4 kcal/mol) and alpha-chymotrypsin (−7.2 kcal/mol) than those observed with tyrosinase, supporting its potential selectivity toward the melanogenic pathway. Furthermore, results from the trypsin inhibition assay showed that the presence of trypsin did not significantly alter the tyrosinase inhibitory activity of the extract (Table 6), indicating that the active components retain their function despite proteolytic exposure. Collectively, these findings suggest that stigmasterol may possess favorable enzymatic stability and selectivity, warranting further investigation for safe topical use in cosmetic or dermatological applications.

**Table 7:** Binding affinities (kcal mol^-1^) of the top eight lead compounds with trypsin and alpha-chymotrypsin.

**Compound name**	**Formula**	**Trypsin** **(2ZQ1) Binding energy (kcal mol^-1^)**	**alpha-Chymotrypsin (4Q2K) Binding energy (kcal mol^-1^)**
Olean-12-ene-3,15,16,21,22,28-hexol, (3β,15α,16α,21β,22α)-	C_30_H_50_O_6_	−7.0	−6.9
5-Chloro-6beta-nitro-5alpha-cholestan-3-one	C_27_H_44_ClNO_3_	−7.0	−7.4
Fenretinide	C_26_H_33_NO_2_	−7.3	−6.5
Cholesterol	C_27_H_46_O	−7.0	−6.5
Stigmasterol	C_29_H_48_O	−7.4	−7.2
Glycodeoxycholic acid	C_26_H_43_NO_5_	−8.2	−7.8
(22S)-21-Acetoxy-6α,11β-dihydroxy-16α,17α-propylmethylenedioxypregna-1,4-diene-3,20-dione	C_27_H_36_O_8_	−8.7	−8.1
N∼2∼-[4-({[3-(Cyclohexylamino)propyl]amino}methyl)benzyl]−6-(1-piperazinyl)-2,4-pyrimidinediamine	C_25_H_40_N_8_	−7.9	−7.3
Control Inhibitors			
(*S*)-N-(4-carbamimidoylbenzyl)-1-(2-(cyclohexylamino)ethanoyl)pyrrolidine-2-carboxamide	C _21_H_31_N_5_O_2_	−8.6	–
(11S)-4,9-dioxo-N-[(2S)-1-oxo-3-phenylpropan-2-yl]−17,22-dioxa-10,30-diazatetracyclo[21.2.2.2∼13,16∼.1∼5,8∼]triaconta-1(25),5,7,13,15,23,26,28-octaene-11-carboxamide	C_36_H_37_N_3_O_6_	–	−8.9
4-[(5-phenyl-1∼{H}-imidazol-2-yl)methylamino]−2-(pyridin-3-ylmethoxy)benzenecarboximidamide	C_23_H_22_N_6_O	–	–

## 4 Discussion

This study employed methanol as the extraction solvent due to its high polarity, enabling the efficient solubilization of a broad spectrum of polar bioactive compounds, including phenolics, alkaloids, and terpenoids, which are associated with antioxidant and anti-tyrosinase activities [[30], [31], [32]]. Its high volatility also facilitates low-temperature removal, minimizing the risk of thermal degradation during concentration [33]. However, methanol maceration presents several limitations. The process is time-consuming, often yields low quantities of target compounds, and can co-extract undesirable substances such as pigments and sugars. Moreover, methanol is toxic and unsuitable for direct use in food or cosmetics without complete removal, and its limited efficiency for extracting non-polar or thermolabile compounds further restricts its application [34].

To overcome these limitations, several advanced green extraction technologies have been developed to improve selectivity, efficiency, and sustainability. Pressurized liquid extraction (PLE) enhances extraction kinetics through elevated temperatures and pressures, reducing solvent consumption and processing time while improving compound recovery, however its application is limited by high equipment costs and the risk of co-extracting matrix contaminants [34]. Ultrasound-assisted extraction (UAE) employs acoustic cavitation to disrupt cellular structures, enhancing mass transfer and extraction efficiency with reduced solvent usage and shorter processing time, although careful control of sonication is required to avoid degradation of sensitive compounds. Microwave-assisted extraction (MAE) utilizes electromagnetic radiation to rapidly heat the solvent matrix system, offering efficient recovery of thermolabile compounds with minimal solvent input, but may encounter challenges related to uniform heating and scale-up for industrial applications [35]. Supercritical fluid extraction (SFE), particularly using CO₂, provides a selective and solvent-free method suitable for lipophilic and heat-sensitive bioactives. It operates at relatively mild conditions, preserves compound integrity, and eliminates solvent residues, making it especially attractive for pharmaceutical and cosmetic use. Nevertheless, SFE exhibits limited efficiency in extracting polar compounds without co-solvents and requires high-pressure systems with considerable cost and technical complexity. In summary, while PLE, UAE, MAE, and SFE offer substantial advantages over traditional methanol maceration in terms of extraction efficiency, compound stability, and environmental compatibility, these approaches also present practical challenges including high capital investment, optimization demands, and limitations in scalability and compound specificity, which must be carefully considered when designing extraction strategies for seaweed-derived bioactive compounds [34,35].

The analysis of 120 chemical components using LC-MS and GC–MS has contributed to establishing the first comprehensive chemical profile of *S. polycystum, C. lentillifera*, and *G. fisheri*. Previous studies on seaweed composition have primarily focused on proximate analyses, including to carbohydrate, protein, mineral content, and fatty acid profiles [36]. In addition, investigation of secondary metabolites such as phenolics, sterols, and terpenoids have largely relied on quantitative phytochemical assays, including total phenolic and flavonoid content measurements, or FTIR analysis [31,32,37,38]. To our knowledge, this is among the first studies to provide detailed chemical profiling of these seaweed species. A comparative analysis based on the presence and absence of the chemical constituents revealed that each of the three seaweed species serves as a rich source of bioactive compounds, with intergroup dissimilarity exceeding 61.1 %. These findings emphasize the distinct chemical profiles of the seaweeds and highlight the diversity of their bioactive compounds, reinforcing their potential significance in pharmaceutical and nutraceutical applications.

The antioxidant and anti-tyrosinase activities of different seaweed species vary considerably, primarily due to differences in their bioactive compound composition and the mechanisms through which these compounds mediate their effects. In this study, the DPPH assay was employed to evaluate antioxidant activity, as this method measures the capacity of compounds to donate hydrogen atoms for free radical neutralization [39]. The results demonstrated a concentration-dependent increase in antioxidant activity, suggesting that the bioactive phytochemicals present in the crude extracts enhance free radical scavenging efficacy at elevated concentrations. Among the seaweeds studied, brown seaweeds exhibited significantly higher antioxidant activity compared to red seaweeds [31,39]. Similarly, in this study, the extract obtained from *S. polycystum* displayed greater antioxidant activity than that of *G. fisheri*, further corroborating the observed trend.

All seaweed species analyzed in this study exhibited higher anti-tyrosinase activity compared to kojic acid, a commonly used standard inhibitor. As shown in Table 8, the anti-tyrosinase activity among different seaweed species ranged from 1.57 to 3981.68 μg mL^-1^ [[40], [41], [42], [43], [44], [45], [46], [47], [48], [49], [50]]. This inhibitory activity can be attributed to the presence of various bioactive compounds, including phenolic compounds, phlorotannin, flavonoids, bromophenols, phytol, and alkaloids. In the present study, the anti-tyrosinase activity ranged from 0.15 to 0.33 µg mL^-1^, which is lower than previously reported valued. Additionally, our findings indicate that the identified compounds exhibited potent and dose-dependent tyrosinase inhibition. Most previous studies on anti-tyrosinase activity have focused on brown seaweed species such as *Ecklonia cava, E. stolonifera, Eisenia bicyclis, Sargassum horneri, S. ilicifolium, S. yendoi* and *Turbinaria conoides* [40,42,44,45,50], as well as red seaweed such as *Grateloupia elliptica* [50]. This study provided the first report on the tyrosinase inhibition activity of *S. polycystum, C. lentilifera* and *G. fisheri*, with activity levels lower than those previously reported for other seaweed species. Among the tested samples, the lipophilic extract of *G. fisheri* exhibited the strongest anti-tyrosinase activity, indicating the presence of potent inhibitory compounds. The retention of tyrosinase inhibition following exposure to trypsin suggests that the active components are resistant to proteolytic degradation, which may enhance their functional stability under physiological conditions. In parallel, molecular docking analyses with trypsin and chymotrypsin revealed that the binding affinities of seaweed-derived compounds were generally moderate and weaker than those observed with tyrosinase. These findings suggest that the compounds exhibit target selectivity, potentially minimizing unintended interactions with common serine proteases. In particular, stigmasterol showed lower binding affinity with trypsin and chymotrypsin compared to tyrosinase, consistent with its potential as a selective tyrosinase inhibitor. The combined enzymatic stability and binding selectivity of stigmasterol suggest its potential for safe and targeted use in dermatological applications.

**Table 8:** Anti-tyrosinase activity across seaweed species.

**Species**	**IC_50_ Values** **(μg mL^-1^ or μM)**	**Significant compounds contributing to tyrosinase inhibition**	**References**
*Bangia fuscopurpurea*	32.4 ± 3.06 μg mL^-1^	Phytol, competitive inhibition	[47]
*Halimeda* spp.	573.00 – 3981.68 μg mL^-1^	Alkaloids, phenols	[41]
			
*Ecklonia cava*	4.38±0.08 μg mL^-1^	Phenolic compounds	[42,50]
*E. stolonifera*	1.57–3.56 μg mL^-1^	974-A, phlorofucofuroeckol-A, eckol	[42]
*Eisenia bicyclis*	4.46±0.52 μg mL^-1^	Phenolic compounds	[50]
*Grateloupia elliptica*	218.77±3.49 μg mL^-1^	Phenolic compounds	[50]
*Ishige okamurae*	8.97±0.91 μg mL^-1^	Phenolic compounds	[50]
*Padina* spp.	132 – 356 μg mL^-1^	Phenolics, flavonoids	[49]
*Sargassum horneri*	6.20±0.22 μg mL^-1^	Phenolic compounds	[50]
*S. ilicifolium*	40.45 μg mL^-1^	Phenolic compounds	[45]
*S. yendoi*	7.15±0.96 μg mL^-1^	Phenolic compounds	[50]
*Sargassum* spp.	47.94 – 650.4 μg mL^-1^	Phenolic compounds, phlorotannins	[46,48]
*Symphyocladia latiuscula*	2.92 – 10.78 μM	Bromophenols	[43]
*Turbinaria conoides*	5.29 μg mL^-1^	Phenolic compounds	[44]
*S. polycystrum*	0.19–4.32 μg mL^-1^	Terpenoid, alkaloids, coumarin	This study
*C. lentilifera*	0.21–0.33 μg mL^-1^	Terpenoid, alkaloids, coumarin	This study
*G. fisheri*	0.15–0.21 μg mL^-1^	Terpenoid, alkaloids, coumarin, stigmasterol	This study

Molecular docking studies further supported the bioactivity of stigmasterol, revealing low binding affinity with both mushroom and human tyrosinase proteins. This strong interaction *in silico* suggests a potential mechanism by which stigmasterol may inhibit tyrosinase activity, warranting further biochemical validation. Additionally, computational toxicity assessments predicted a favorable safety profile for stigmasterol, suggesting its potential suitability for further investigation in pharmaceutical and cosmetic applications.

Stigmasterol, an unsaturated phytosterol categorized within the tetracyclic triterpene class, is commonly present in various plants [51]. In seaweed, its presence varies depending on species, geographical distribution, and environmental conditions. Stigmasterol has been identified across different seaweed taxa, including red, brown, and green macroalgae. Among red seaweed, species such as *Gelidium spinosum, Palmaria decipiens*, and *Pyropia endiviifolia* have been reported to contain stigmasterol [52,53]. Brown seaweed such as *Adenocystis utricularis* and *Desmarestia antarctica* also exhibit stigmasterol in their composition [54], with *Saccharina latissima* and *Macrocystis pyrifera* similarly demonstrating significant levels of this sterol [55,56]. Green seaweed, including *Chaetomorpha basiretorsa* and *Ulva lactuca*, have been analyzed for sterol content, with stigmasterol identified as a minor but significant component [56,57].

Previous studies have demonstrated that stigmasterol exhibits cytotoxic activity, particularly in regulating cell proliferation and inflammation, supporting its potential therapeutic application. Its broad bioactivity includes anticancer, antidiabetic, and anti-inflammatory properties, with IC_50_ values varying based on the target cells or enzymes. In breast cancer cells (MCF-7), stigmasterol exhibited potent cytotoxic activity, with an IC_50_ value of 0.1623 µM, significantly lower than that of cisplatin (13.2 µM), indicating its highly efficacy as an anticancer agent [58]. In antidiabetic studies, stigmasterol isolated from *Morinda citrifolia* achieved an IC_50_ of 10.29 ± 0.76 µg mL^-1^ in inhibiting human α-amylase activity, outperforming both the fruit extract and the standard drug acarbose [59]. In the context of this study, stigmasterol demonstrated an IC_50_ value of 3.38 ± 0.28 µg mL^-1^ in HaCaT cells, showing significant cytotoxicity compared to the crude *G. fisheri* extract (IC_50_ = 161.00±23.13 µg mL^-1^). Despite its cytotoxic potency, stigmasterol was non-toxic to HaCaT cells at concentrations as low as 0.006 µg mL^-1^, supporting its safety in cosmetic formulations for skin health. The variability in IC_50_ values across different systems underscores stigmasterol’s diverse bioactivity, which is concentration- and target-specific. While its strong anticancer and antidiabetic effects suggest its therapeutic potential, its cytotoxicity in skin cells highlights careful optimization of concentrations in formulations. Although the crude extract exhibits lower cytotoxicity, it may still offer bioactive benefits due to its complex composition. The observed tyrosinase inhibition and cytotoxicity profile of stigmasterol suggest its potential for further investigation in dermatological applications, especially in addressing pigmentation disorders. Further research should explore stigmasterol's mechanism of action to better understand its bioactivity spectrum and ensure its safe application in dermatological and pharmaceutical products.

Beyond dermatological applications, stigmasterol exhibits significant promise in pharmaceuticals and nutraceuticals due to its diverse bioactive properties. Its antidiabetic activity supports its potential as a functional food ingredient or dietary supplement [53]. Additionally, its antibacterial and antifouling properties highlight its suitability as a natural compound for industrial applications [60]. Recent research has explored its anti-tyrosinase activity of stigmasterol, particularly in combination with other compounds. A mixture of β-sitosterol and stigmasterol has demonstrated notable tyrosinase inhibitory effects, suggesting a potential role for stigmasterol in pigmentation control [61]. However, the specific role of stigmasterol as an independent tyrosinase inhibitor remains underexplored, with existing studies primarily focusing on combined effects. Our computational studies reveal that stigmasterol exhibits strong binding interaction with tyrosinase enzymes from both mushroom and human sources, supporting its role as a tyrosinase inhibitor. Additionally, cell toxicity assays show that pure stigmasterol is non-toxic to HaCaT cells at low concentration and promotes cell proliferation. These findings provide valuable insights into its potential as a standalone tyrosinase inhibitor, supporting its application in cosmetic formulations for skin whitening and hyperpigmentation treatment. The natural origin of stigmasterol presents a safer alternative to synthetic tyrosinase inhibitors [61,62]. Incorporating natural tyrosinase inhibitors such as stigmasterol from seaweed into cosmetic formulations ensures both efficacy and safety while meeting hygienic and regulatory standards. Furthermore, these findings highlight the importance of seaweed extracts as a sustainable source of bioactive compounds for cosmetic applications.

## 5 Conclusion

This study provides comprehensive insights into the bioactive potential of hydrophilic and lipophilic extracts from three seaweed species including *S. polycystum, C. lentillifera*, and *G. fisheri*, with distinct chemical compositions identified through LC-MS and GC–MS profiling. Among these, the lipophilic extract of *G. fisheri* exhibited the strongest anti-tyrosinase activity. Stigmasterol demonstrated strong *in silico* binding affinity, favorable skin permeability, and low cytotoxicity in HaCaT cells, suggesting its potential as a safe and effective tyrosinase inhibitor. Its affordability and scalability further enhance its appeal for dermatological formulations targeting hyperpigmentation. Additionally, the observed proteolytic stability and selective interaction profile of stigmasterol suggest its safety and functional compatibility for topical application. Future research should focus on optimizing sustainable extraction methods, advancing formulation development, and conducting preclinical and clinical evaluations to support the application of seaweed-derived compounds in cosmeceutical products.

## CRediT authorship contribution statement

**Arachaporn Thong-olran:** Writing – original draft, Visualization, Validation, Software, Methodology, Investigation, Data curation. **Supatchar Sermsakulwat:** Writing – original draft, Visualization, Software, Methodology, Investigation. **Tiwtawat Napiroon:** Writing – original draft, Validation, Resources, Methodology, Funding acquisition, Conceptualization. **Phuphiphat Jaikaew:** Writing – original draft, Validation, Funding acquisition. **Sumet Kongkiatpaiboon:** Validation, Software, Funding acquisition. **Ngampuk Tayana:** Writing – original draft, Validation, Software, Funding acquisition. **Bongkot Wichachucherd:** Validation, Resources, Funding acquisition. **Theppanya Charoenrat:** Writing – original draft, Visualization, Validation, Resources, Methodology, Funding acquisition, Conceptualization. **Thrissawan Traijitt:** Validation, Methodology. **Supenya Chittapun:** Writing – review & editing, Writing – original draft, Visualization, Validation, Supervision, Software, Resources, Project administration, Methodology, Funding acquisition, Data curation, Conceptualization.

## Declaration of competing interest

The authors declare that they have no known competing financial interests or personal relationships that could have appeared to influence the work reported in this paper.

## Data Availability

Data will be made available on request.

## Appendix Supplementary materials

Image, application 1

Image, application 2

Image, application 3

Image, application 4

## References

[1] Morais T., Cotas J., Pacheco D., Pereira L. (2021). Seaweeds compounds: an ecosustainable source of cosmetic ingredients?. Cosmetics.

[2] Talreja S., Tiwari S. (2024). Revelation the Sea’s secret: seaweed’s rise as a potent cosmetic ingredient. Int. J. Biol. Pharm. Sci. Arch..

[3] H.S. Kalasariya, V.K. Yadav, K.K. Yadav, V. Tirth, A. Algahtani, S. Islam, N. Gupta, B.-H. Jeon, Seaweed-based molecules and their potential biological activities: an eco-sustainable cosmetics, Molecules 26(17) (2021) 5313, 10.3390/molecules26175313. PMC8434260 34500745

[4] Kalasariya H.S., Pereira L. (2022). Dermo-cosmetic benefits of marine macroalgae-derived phenolic compounds. Appl. Sci..

[5] Malakar B., Mohanty K., Mandotra S.K., Upadhyay A.K., Ahluwalia A.S. (2021). The Budding Potential of Algae in Cosmetics.

[6] dos Santos T.C., Machado L.P., Santos A., Martins R.C.C., Cavalcanti D.N., Bueno G.W., Sanches A.L.M., Obando J.M.C. (2024). Dermocosmetic properties of bioproducts from *Sargassum* Macroalgae: chemical aspects, challenges, and opportunities. Front. Mar. Sci..

[7] Reyes E.D.L., Basaran A. (2020). Bioactive properties of *Sargassum siliquosum* J. Agardh (Fucales, Ochrophyta) and its potential as source of skin-lightening active ingredient for cosmetic application. J. Appl. Pharm. Sci..

[8] Kalasariya H.S., Pereira L., Patel N.B. (2023). Comprehensive phytochemical analysis and bioactivity evaluation of *Padina boergesenii*: unveiling its prospects as a promising cosmetic component. Mar. Drugs.

[9] López-Hortas L., Flórez-Fernández N., Torres M.D., Ferreira-Anta T., Casas M.P., Balboa E.M., Falqué E., Domínguez H. (2021). Applying seaweed compounds in cosmetics, cosmeceuticals and nutricosmetics. Mar. Drugs.

[10] Salehi B., Sharifi-Rad J., Seca A.M.L., Pinto D.C.G.A., Michalak I., Trincone A., Mishra A.P., Nigam M., Zam W., Martins N. (2019). Current trends on seaweeds: looking at chemical composition, phytopharmacology, and cosmetic applications. Molecules.

[11] Aminina N.M., Karaulova E.P., Vishnevskaya T.I., Yakush E.V., Kim Y.-K., Nam K.-H., Son K.-T. (2020). Characteristics of polyphenolic content in brown algae of the Pacific Coast of Russia. Molecules.

[12] Sadeghi A., Rajabiyan A., Nezhad N.M., Nabizade N., Alvani A., Zarei-Ahmady A.A. (2024). A review on Persian Gulf brown algae as potential source for anticancer drugs. Algal Res.

[13] Abbott I.A. (1999).

[14] Dawson E.Y. (1954). Notes on tropical Pacific marine algae. Bull. South. Calif. Acad. Sci..

[15] Egerod L.E. (1975). Marine algae of the Andaman Sea Coast of Thailand: chlorophyceae. Bot. Mar..

[16] Huisman J.M. (2000).

[17] K. Lewmanomont, H. Ogawa, Common Seaweeds and Seagrasses of Thailand.

[18] Zarei M., Ebrahimpour A., Abdul-Hamid A., Anwar F., Bakar F.A., Philip R., Saari N. (2014). Identification and characterization of papain-generated antioxidant peptides from palm kernel cake proteins. Food Res. Int..

[19] Liang C.P., Chang C.H., Liang C.C., Hung K.Y., Hsieh C.W. (2014). Vitro antioxidant activities, free radical scavenging capacity, and tyrosinase inhibitory of flavonoid compounds and ferulic acid from *Spiranthes sinensis*(Pers.) Ames. Molecules.

[20] Yu Q., Fan L., Duan Z. (2019). Five individual polyphenols as tyrosinase inhibitors: inhibitory activity, synergistic effect, action mechanism, and molecular docking. Food Chem..

[21] Mughal E.U., Ashraf J., Hussein E.M., Nazir Y., Alwuthaynani A.S., Naeem N., Sadiq A., Alsantali R.I., Ahmed S.A. (2022). Design, synthesis, and structural characterization of thioflavones and thioflavonols as potential tyrosinase inhibitors: in Vitro and In Silico studies. ACS Omega.

[22] Veerichetty V., Saravanabavan I. (2023). Molecular docking study of nuciferine as a tyrosinase inhibitor and its therapeutic potential for hyperpigmentation. Genomics Inform.

[23] Chatatikun M., Tedasen A., Pattaranggoon N.C., Palachum W., Chuaijit S., Mudpan A., Pruksaphanrat S., Sohbenalee S., Yamasaki K., Klangbud W.K. (2023). Antioxidant activity, anti-tyrosinase activity, molecular docking studies, and molecular dynamic simulation of active compounds found in Nipa palm vinegar. PeerJ.

[24] O’Boyle N.M., Banck M., James C.A., Morley C., Vandermeersch T., Hutchison G.R. (2011). Open Babel: an Open chemical toolbox. J. Cheminform..

[25] Pushpalatha N., Ramadevi N., Kishore S.C., Bellucci S. (2023). Computational-simulation-based behavioral analysis of chemical compounds. J. Compos. Sci..

[26] Morris G.M., Huey R., Olson A.J. (2008). Using AutoDock for ligand-receptor docking. Curr. Protoc. Bioinformatics Chapter.

[27] Pires D.E., Blundell T.L., Ascher D.B. (2015). pkCSM: predicting small-molecule pharmacokinetic and toxicity properties using graph-based signatures. J. Med. Chem..

[28] Sethy R., Kullu B. (2022). Micropropagation of ethnomedicinal plant calotropis sp. and enhanced production of Stigmasterol. Plant Cell Tiss. Organ Cult..

[29] Takatsuto S., Ikekawa N. (1987). Synthesis of 5α-cholestan-6-one derivatives with some substituents at the C-1, C-2, or C-3 position. ChemInform.

[30] Lee Z.J., Xie C., Duan X., Ng K., Suleria H.A.R. (2024). Optimization of ultrasonic extraction parameters for the recovery of phenolic compounds in brown seaweed: comparison with conventional techniques. Antioxidants.

[31] Sobuj M.K.A., Islam M.A., Islam M.S. (2021). Effect of solvents on bioactive compounds and antioxidant activity of *Padina tetrastromatica* and *Gracilaria tenuistipitata* seaweeds collected from Bangladesh. Sci. Rep..

[32] Lee H.-H., Kim J.-S., Jeong J.-H., Park S.M., Sathasivam R., Lee S.Y., Kim C.S. (2022). Effect of different solvents on the extraction of compounds from different parts of *Undaria pinnatifida* (Harvey) Suringar. J. Mar. Sci. Eng..

[33] Anoor P.K., Yadav A.N., Rajkumar K., Kande R., Tripura C., Naik K.S., Burgula S. (2022). Methanol extraction revealed anticancer compounds quinic acid, 2(5H)-furanone and phytol in *Andrographic paniculatra*. Mol. Clin. Oncol..

[34] Perez-Vazquez A., Carpena M., Barciela P., Cassani L., Simal-Gandara J., Prieto M.A. (2023). Pressurized liquid extraction for the recovery of bioactive compounds from seaweeds for food industry application: a review. Antioxidants.

[35] Quitério E., Grosso C., Ferraz R., Delerue-Matos C., Soares C. (2023). A critical comparison of the advanced extraction techniques applied to obtain health-promoting compounds form seaweeds. Mar. Drugs..

[36] Premarathna A.D., Tuvikene R., Fernando P.H.P. (2022). Comparative analysis of proximate compositions, mineral and functional chemical groups of 15 different seaweed species. Sci. Rep..

[37] El-Said G.F., El-Sikaily A. (2013). Chemical composition of some seaweed from Mediterranean Sea Coast, Egypt. Environ. Monit. Assess..

[38] Ertani A., Francioso O., Tinti A., Schiavon M., Pizzeghello D., Nardi S. (2018). Evaluation of seaweed extracts from *Laminaria* and *ascophyllum nodosum* spp. As biostimulants in *Zea mays* L. Using a combination of chemical, biochemical and morphological approaches. Front. Plant Sci..

[39] Hejna M., Dell’Anno M., Liu Y. (2024). Assessment of the antibacterial and antioxidant activities of seaweed-derived extracts. Sci. Rep..

[40] Kang H.S., Kim H.R., Byun D.S. (2004). Tyrosinase inhibitors isolated from the edible brown Alga *Ecklonia stolonifera*. Arch. Pharm. Res..

[41] M. Gazali, Aktivitas inhibitor tirosinase Rumput Laut *Halimeda* spp Dari Pesisir Aceh Barat, J. Pengolahan Teknol., 10.35308/JPT.V5I2.1034.

[42] Manandhar B., Wagle A., Seong S.H., Paudel P., Kim H.R., Jung H.A., Choi J.S. (2019). Phlorotannins with potential anti-tyrosinase and antioxidant activity isolated from the marine seaweed *ecklonia stolonifera*. Antioxidants.

[43] Paudel P., Wagle A., Seong S.H., Park H.J., Jung H.A., Choi J.S. (2019). A new tyrosinase inhibitor from the red Alga *Symphyocladia latiuscula* (Harvey) Yamada (Rhodomelaceae). Mar. Drugs.

[44] Sari D.M., Anwar E., Nurjanah A.E.Arifianti (2019). Antioxidant and tyrosinase inhibitor activities of ethanol extracts of brown seaweed (*Turbinaria conoides*) as lightening ingredient. Pharmacogn. J..

[45] Arguelles E.D.L.R. (2021). Evaluation of antioxidant capacity, tyrosinase inhibition, and antibacterial activities of Brown seaweed, *Sargassum ilicifolium* (Turner) C. Agardh 1820 for cosmeceutical application. J. Fish. Environ..

[46] Landa-Cansigno C., Serviere-Zaragoza E., Morales-Martínez T.K., Ascacio-Valdes J.A., Morreeuw Z.P., Gauyat C., Stiger-Pouvreau V., Reyes A.G. (2023). The antioxidant and anti-elastase activity of the brown seaweed *sargassum horridum* (Fucales, Phaeophyceae) and their early phenolics and saponins profiling for green cosmetic applications. Algal Res.

[47] Wang X., Chang G., Xu Y., Li Z., Du X.P., Yang Y., Jiang Z., Ni H., Li Q. (2023). Effect of phytol isolated from edible red alga (*Bangia fusco-purpurea*) on tyrosinase inhibition and its application on food preservation. LWT - Food Sci. Technol..

[48] Abubakar M., Setyaningsih I., Bayu A., Desniar D. (2024). Antioxidant activity and tyrosinase of brown seaweed extract using ultrasonic and magnetic stirrer extraction methods. Int. J. Chem. Biochem. Sci..

[49] Akbar A.N., Rasyid H., Natsir H., Bahrun B., Soekamto N.H. (2024). Tyrosinase inhibitory activity of *n*-hexane, ethyl acetate and methanol extracts of *padina* sp. Res. J. Pharm. Technol..

[50] Ryu J.W., Yim M.J., Kim J.Y., Lee J.M., Lee M.S., Lee D.S., Hwang J.Y., Kim K.T., Kim Y.M., Eom S.H. (2024). Tyrosinase inhibition effects of Korean edible brown. Green, and Red Seaweed Extracts, Fish. Aquat. Sci..

[51] Adeniran L., Zaifada A., Olorunmonu B., Lawal R. (2024). Isolation and characterization of stigmasterol from *Psidium guajava* leaves: a promising bioactive compound with therapeutic potential. Open Access J. Microbiol. Biotechnol..

[52] de Santi I.I., Pacheco B.S., Venzke D., Freitag R.A., de Almeida L.S., Colepicolo P., Fujii M.T., Dias D., Pereira C.M.P. (2021). Sterols in red macroalgae from Antarctica: extraction and quantification by gas chromatography–Mass spectrometry. Polar Biol..

[53] Poulose N., Sajayan A., Ravindran A., Chandran A., Priyadharshini G.B., Selvin J., Kiran G.S. (2021). Anti-diabetic potential of a stigmasterol from the seaweed *gelidium spinosum* and its application in the formulation of nanoemulsion conjugate for the development of functional biscuits. Front. Nutr..

[54] Pereira C.M.P., Nunes C.F.P., Zambotti-Villela L., Streit N.M., Dias D., Pinto E., Gomes C.B., Colepicolo P., Colepicolo P. (2017). Extraction of sterols in brown macroalgae from Antarctica and their identification by liquid chromatography coupled with tandem mass spectrometry. J. Appl. Phycol..

[55] de Jong D.L.C., Timmermans K.R., de Winter J.M., Derksen G.C.H. (2021). Effects of nutrient availability and light intensity on the sterol content of *Saccharina latissima* (Laminariales, Phaeophyceae). J. Appl. Phycol..

[56] Serviere-Zaragoza E., Hurtado-Oliva M.Á., Mazariegos-Villarreal A., Arjona O., Palacios E. (2021). Seasonal and interannual variation of sterols in macrophytes from the Pacific Coast of Baja California Peninsula (Mexico). Phycol. Res..

[57] Shi D., Fan X., Sun J., Han L., Shi J. (2008). Steroids from green Alga *Chaetomorpha basiretorsa* Setchell. Chin. J. Oceanol. Limnol..

[58] Sianipar N.F., Hadisaputri Y.E., Assidqi K., Salam S., Yusuf M., Destiarani W., Purnamaningsih R., So I.G. (2021). Characterization and investigation of stigmasterol isolated from rodent tuber mutant plant (*Typhonium flagelliforme*), its molecular docking as anticancer on MF-7 cells. Preprints.

[59] Lolok N., Sumiwi S.A., Sahidin I., Levita J. (2023). Stigmasterol isolated from the ethyl acetate fraction of *morinda citrifolia* fruit (Using the Bioactivity Guided Method) inhibits α-amylase activity: in vitro and in vivo analyses. World Acad. Sci. J..

[60] Bakar K., Mohamad H., Tan H.S., Latip J. (2019). Sterols compositions, antibacterial, and antifouling properties from two Malaysian seaweeds: *dictyota dichotoma* and *sargassum granuliferum*. J. Appl. Pharm. Sci..

[61] Muhammad A., Idris M.M., Ali U., Umar A., Sirat H. (2023). Characterization and tyrosinase activities of a mixture of β-sitosterol and stigmasterol from *Bauhinia rufescens* Lam. Acta Pharm. Indones..

[62] Pylypenko L., Sevastyanova E., Novikova N., Makovska Т., Kilimenchuk H. (2019). The safe transdermal cosmetic product with antityrosinase activity. Food Sci. Technol. Int..

