# Supplementary material for: Seaweed-derived bioactives with anti-tyrosinase activity: a potential for skin-whitening cosmetics with in silico and in vitro approaches
Source: Biotechnol Rep (Amst). 2025 Aug 7;47:e00910. doi: 10.1016/j.btre.2025.e00910 (PMC12357141; doi:10.1016/j.btre.2025.e00910)
Supplement: Supplementary file 1 [file mmc1.docx]

**Supplementary Table 1.** Relative front (Rf) values of lipophilic extracts in solvent system.

**Supplementary Table 2.** List of chemical composition detected from LC-MS from the three seaweed species (S: *S. polycystum*, C: *C. lentillifera*, G: *G. fisheri*)

**Supplementary Table 3.** List of chemical composition detected from GC-MS from the three seaweed species (S: *S. polycystum*, C: *C. lentillifera*, G: *G. fisheri*)

**Supplementary Table 1.** Relative front (Rf) values of lipophilic extracts in solvent system.

| Seaweed Species | R_f values_ | | |
| --- | --- | --- | --- |
|  | Terpenoid | Alkaloids | Coumarin |
| Lipophilic extracts of *S. polycystum* | 0.26 | 0.58 | 0.68 |
| Lipophilic extracts of *C. lentillifera* | 0.26 | 0.58 | 0.68 |
| Lipophilic extracts of *G. fisheri* | 0.26 | 0.58, 0.90, base line | 0.68 |
| Hydrophilic extracts of *S. polycystum* | 0.57 | 0.24 | - |
| Hydrophilic extracts of *C. lentillifera* | 0.50 | - | - |
| Hydrophilic extracts of *G. fisheri* | 0.54 | - | - |

**Supplementary Table 2.** List of chemical composition detected from LC-MS from the three seaweed species (S: *S. polycystum*, C: *C. lentillifera*, G: *G. fisheri*)

| No. | Name | Formula | RT (min) | Reference Ion | Molecular Weight | m/z | Seaweeds | | |
| --- | --- | --- | --- | --- | --- | --- | --- | --- | --- |
|  |  |  |  |  |  |  | S | C | G |
| 1 | C16 phytosphingosine | C_16_H_35_ NO_3_ | 4.443 | [M+H]+1 | 289.26162 | 290.26889 | *🗸* | *🗸* | *🗸* |
| 2 | N~2~-[4-({[3-(Cyclohexylamino)propyl]amino}methyl)benzyl]-6-(1-piperazinyl)-2,4-pyrimidinediamine | C_25_H_40_ N_8_ | 4.494 | [M+H]+1 | 452.33616 | 453.34342 | *🗸* | *🗸* | *🗸* |
| 3 | (3S,5R,8R,9R,10R,12R,13R,14R,17S)-17-[(2R)-2,6-Dihydroxy-6-methyl-2-heptanyl]-4,4,8,10,14-pentamethylhexadecahydro-1H-cyclopenta[a]phenanthrene-3,12-diyl (2S,2′S)bis[4-methyl-2-({[(2-methyl-2-propanyl )oxy]carbonyl}amino)pentanoate] (non-preferred name) | C_52_H_92_ N_2_O_10_ | 5.119 | [M+2H]+2 | 904.67238 | 453.34347 | *🗸* | *🗸* | *🗸* |
| 4 | 2,6-Dimethoxy-4-propylphenol | C_16_H_35_ NO_3_ | 0.35 | [2M+Na]+1 | 196.11051 | 415.2115 | *🗸* | *🗸* | *🗸* |
| 5 | Thiamine | C_25_H_40_ N_8_ | 7.554 | [M+H]+1 | 264.10475 | 265.11203 |  | *🗸* | *🗸* |
| 6 | Tropolone A | C_52_H_92_ N_2_O_10_ | 0.339 | [M+H]+1 | 431.23094 | 432.23822 | *🗸* | *🗸* |  |
| 7 | Acetylcholine | C_16_ H_35_NO_3_ | 5.929 | [M+H]+1 | 145.11022 | 146.11754 | *🗸* |  | *🗸* |
| 8 | Diethanolamine | C_25_H_40_ N_8_ | 6.097 | [M+H]+1 | 105.07889 | 106.08617 | *🗸* | *🗸* |  |

**Supplementary Table 3.** List of chemical composition detected from GC-MS from the three seaweed species (S: *S. polycystum*, C: *C. lentillifera*, G: *G. fisheri*)

| Bioactive Compounds from Seaweed | | | | | | |
| --- | --- | --- | --- | --- | --- | --- |
| No. | Name | Molecular formula | Seaweed | | | PubChem CID |
|  |  |  | S | C | G |  |
| 1 | Methyl 13,16-docosadienoate (cis-13,16-Docosadienoic acid methyl ester) | C_23_H_42_O_2_ | 🗸 |  |  | 15748573 |
| 2 | 1-(3,6,6-Trimethyl-1,6,7,7a-tetrahydrocyclopenta[c]pyran-1-yl) ethanone | C_13_H_18_O_2_ | 🗸 |  |  | 605654 |
| 3 | Cyclopropane butanoic acid, 2-[[2-[[2-[(2-pentylcyclopropyl) methyl] cyclopropyl] methyl] cyclopropyl] methyl]-, methyl ester | C_25_H_42_O_2_ | 🗸 | 🗸 |  | 554084 |
| 4 | Octahydrobenzo[b]pyran, 4a-acetoxy-5,5,8a-trimethyl- | C_14_H_24_O_3_ | 🗸 |  |  | 574096 |
| 5 | Propanoic acid, 2-(3-acetoxy-4,4,14-trimethylandrost-8-en-17-yl)- | C_27_H_42_O_4_ | 🗸 | 🗸 | 🗸 | 631957 |
| 6 | 5,6,6-Trimethyl-5-(3-oxobut-1-enyl)-1-oxaspiro [2.5] octan-4-one | C_14_H_20_O_3_ | 🗸 |  |  | 5363139 |
| 7 | Tridecanoic acid, 12-methyl-, methyl ester (Methyl 12-methyltridecanoate) | C_15_H_30_O_2_ | 🗸 | 🗸 | 🗸 | 21204 |
| 8 | Methyl tetradecanoate | C_15_H_30_O_2_ | 🗸 | 🗸 |  | 31284 |
| 9 | 6-Hydroxy-4,4,7a-trimethyl-5,6,7,7a-tetrahydrobenzofuran-2(4H)-one | C_11_H_16_O_3_ | 🗸 | 🗸 | 🗸 | 14334 |
| 10 | 9α-Fluoro-17α-methyl-4-androsten-3α, 6β,11β,17β-tetra-ol (h_35_Fluoxymesterone-m) | C_20_H_31_FO_4_ | 🗸 | 🗸 |  | 154585430 |
| 11 | Methyl 13-methyltetradecanoate | C_16_H_32_O_2_ | 🗸 | 🗸 |  | 5077204 |
| 12 | Tetradecanoic acid, 12-methyl-, methyl ester (Methyl 12-methyltetradecanoate) | C_16_H_32_O_2_ | 🗸 | 🗸 |  | 21206 |
| 13 | Pentadecanoic acid, methyl ester (Methyl pentadecanoate) | C_16_H_32_O_2_ | 🗸 |  |  | 23518 |
| 14 | 2,3-Dimethoxy-5-methyl-6-dekaisoprenyl-chinon | C_59_H_90_O_4_ | 🗸 |  |  | 5366029 |
| 15 | 2-Pentadecanone, 6,10,14-trimethyl- (6,10,14-Trimethylpentadecan-2-one) | C_18_H_36_O | 🗸 | 🗸 |  | 10408 |
| 16 | 13-Heptadecyn-1-ol | C_17_H_32_O | 🗸 |  | 🗸 | 557439 |
| 17 | cis-10-Nonadecenoic acid | C_19_H_36_O_2_ | 🗸 |  |  | 5312513 |
| 18 | 9-Hexadecenoic acid, methyl ester, (Z)- (Methyl palmitoleate) | C_17_H_32_O_2_ | 🗸 |  | 🗸 | 643801 |
| 19 | Hexadecanoic acid, methyl ester (Methyl palmitate) | C_17_H_34_O_2_ | 🗸 | 🗸 | 🗸 | 8181 |
| 20 | Phthalic acid, butyl dodecyl ester | C_24_H_38_O_4_ | 🗸 |  |  | 96361 |
| 21 | n-Hexadecanoic acid (Palmitic acid) | C_16_H_32_O_2_ | 🗸 | 🗸 | 🗸 | 985 |
| 22 | Estra-1,3,5(10)-trien-17β-ol (Estra-1(10),2,4-trien-17-ol) | C_18_H_24_O | 🗸 | 🗸 |  | 13058340 |
| 23 | Oleic Acid | C_18_H_34_O_2_ | 🗸 | 🗸 |  | 445639 |
| 24 | Ethanol, 2-(9-octadecenyloxy)-, (Z)- (Emulphor) | C_20_H_40_O_2_ | 🗸 | 🗸 | 🗸 | 5364713 |
| 25 | Ursodeoxycholic acid | C_24_H_40_O_4_ | 🗸 |  |  | 31401 |
| 26 | 9,10-Secocholesta-5,7,10(19)-triene-3,24,25-triol, (3β,5Z,7E)- (Secalciferol) | C_27_H_44_O_3_ | 🗸 | 🗸 |  | 5283748 |
| 27 | 9,12-Octadecadienoic acid (Z,Z)-, methyl ester (Methyl octadecadienoate) (Methyl linoleate) | C_19_H_34_O_2_ | 🗸 | 🗸 |  | 5284421 |
| 28 | 9-Octadecenoic acid, methyl ester, (E)- (Methyl (9E)-9-octadecenoate) (Methyl elaidate) | C_19_H_36_O_2_ | 🗸 |  |  | 5280590 |
| 29 | 12-Octadecenoic acid, methyl ester | C_19_H_36_O_2_ | 🗸 |  |  | 5364503 |
| 30 | Heptadecanoic acid, 16-methyl-, methyl ester (Methyl isostearate) | C_19_H_38_O_2_ | 🗸 |  |  | 110444 |
| 31 | 5,8,11,14-Eicosatetraenoic acid, methyl ester, (all-Z)- (MFCD00016775) (Methyl arachidonate) | C_21_H_34_O_2_ | 🗸 |  | 🗸 | 6421258 |
| 32 | Docosahexaenoic acid, 1,2,3-propanetriyl ester (Glyceryl tridocosahexaenoate) | C_69_H_98_O_6_ | 🗸 |  | 🗸 | 9546569 |
| 33 | 7,10,13-Eicosatrienoic acid, methyl ester (Methyl eicosa-7,10,13-trienoate) | C_21_H_36_O_2_ | 🗸 |  | 🗸 | 5365659 |
| 34 | 9,12,15-Octadecatrienoic acid, 2,3-dihydroxypropyl ester, (Z,Z,Z)- (1-Monolinolenoyl-rac-glycerol) | C_21_H_36_O_4_ | 🗸 |  |  | 5367328 |
| 35 | Ethyl iso-allocholate (Ethyl cholate) | C_26_H_44_O_5_ | 🗸 | 🗸 | 🗸 | 6452096 |
| 36 | 1-Heptatriacontanol | C_37_H_76_O | 🗸 | 🗸 | 🗸 | 537071 |
| 37 | Methyl 9,12-epithio-9,11-octadecanoate (2-Thiopheneoctanoic acid, 5-hexyl-, methyl ester) | C_19_H_32_O_2_S | 🗸 |  |  | 602206 |
| 38 | 11-Eicosenoic acid, methyl ester | C_21_H_40_O_2_ | 🗸 |  |  | 5319603 |
| 39 | Oxiraneoctanoic acid, 3-octyl-, cis- (Epoxyoleic acid) | C_18_H_34_O_3_ | 🗸 |  |  | 119250 |
| 40 | 6-Methyl-11-propenyl-5-(toluene-4-sulfonyloxy)-12,13-dioxatricyclo[7.3.1.0(1,6)]tridecane-8-carboxylic acid, methyl ester | C_24_H_32_O_7_S | 🗸 |  |  | 5368834 |
| 41 | Cholestan-3-one, cyclic 1,2-ethanediyl acetal, (5β)-  (Cholestan-3-one, cyclic 1,2-ethanediyl acetal, (5alpha)-) | C_29_H_50_O_2_ | 🗸 |  |  | 567452 |
| 42 | 2-Deoxychamaedroxide | C_20_H_22_O_6_ | 🗸 |  |  | 129008869 |
| 43 | 13-Docosenoic acid, methyl ester (Methyl (13E)-13-docosenoate) | C_23_H_44_O_2_ | 🗸 |  |  | 5363109 |
| 44 | Hexadecanoic acid, 1-(hydroxymethyl)-1,2-ethanediyl ester (1,2-Dipalmitoyl-rac-glycerol) | C_35_H_68_O_5_ | 🗸 | 🗸 | 🗸 | 99931 |
| 45 | B(9a)-Homo-19-norpregna-9(11),9a-dien-20-one, 3-(dimethylamino)-4,4,14-trimethyl-, (3β,5α)- | C_26_H_41_NO | 🗸 | 🗸 |  | 551567 |
| 46 | 7,8-Epoxylanostan-11-ol, 3-acetoxy- | C_32_H_54_O_4_ | 🗸 |  |  | 541562 |
| 47 | Bufa-20,22-dienolide, 3-(acetyloxy)-14,15-epoxy-16-hydroxy-, (3β,5β,15β,16β)- | C_26_H_34_O_6_ | 🗸 |  |  | 70693000 |
| 48 | (22S)-6α,11β,21-Trihydroxy-16α,17α-propylmethylenedioxypregna-1,4-diene-3,20-dione | C_25_H_34_O_7_ | 🗸 |  |  | 633585 |
| 49 | 1H-Cyclopropa[3,4]benz[1,2-e]azulene-4a,5,7b,9,9a(1aH)-pentol, 3-[(acetyloxy)methyl]-1b,4,5,7a,8,9-hexahydro-1,1,6,8-tetramethyl-, 5,9,9a-triacetate, [1aR-(1aα,1bβ,4aβ,5β,7aα,7bα,8α,9β,9aα)]- (Taxuspin F) | C_28_H_38_O_10_ | 🗸 |  | 🗸 | 10347049 |
| 50 | Cholest-5-en-3-one | C_27_H_44_O | 🗸 |  | 🗸 | 9908107 |
| 51 | Stigmasta-5,24(28)-dien-3-ol,(3β,24Z)- (Stigmasta-5,24-dien-3beta-ol) | C_29_H_48_O | 🗸 |  |  | 5378817 |
| 52 | Pregn-4-ene-3,20-dione, 11-hydroxy-, (11α)- | C_21_H_30_O_3_ | 🗸 |  |  | 92750 |
| 53 | Stigmasterol | C_29_H_48_O | 🗸 |  | 🗸 | 5280794 |
| 54 | Astaxanthin | C_40_H_52_O_4_ | 🗸 | 🗸 |  | 5281224 |
| 55 | Erucic acid | C_22_H_42_O_2_ | 🗸 |  |  | 5281116 |
| 56 | Strobilactone A | C_15_H_22_O_4_ |  | 🗸 |  | 23955806 |
| 57 | Albocycline | C_18_H_28_O_4_ |  | 🗸 | 🗸 | 6440973 |
| 58 | Azuleno[4,5-b]furan-2,9-dione, decahydro-6a-hydroxy-6,9a-dimethyl-3-methylene-, [3aS-(3a.α.,6.β.,6a.α.,9a.β.,9b.α)]- (Coronopolin) | C_15_H_20_O_4_ |  | 🗸 |  | 257278 |
| 59 | 2-[4-Methyl-6-(2,6,6-trimethylcyclohex-1-enyl)hexa-1,3,5-trienyl]cyclohex-1-en-1-carboxaldehyde | C_23_H_32_O |  | 🗸 |  | 5363101 |
| 60 | Fenretinide | C_26_H_33_NO_2_ |  | 🗸 | 🗸 | 5288209 |
| 61 | Cumanin | C_15_H_22_O_4_ |  | 🗸 |  | 353378 |
| 62 | Dihydroxanthin | C_17_H_24_O_5_ |  | 🗸 |  | 536922 |
| 63 | Propiolic acid, 3-(1-hydroxy-2-isopropyl-5-methylcyclohexyl)-, ethyl ester | C_15_H_24_O_3_ |  | 🗸 |  | 534254 |
| 64 | 10,12,14-Nonacosatriynoic acid (15,17,19-Nonacosatriynoic acid) | C_29_H_46_O_2_ |  | 🗸 |  | 583042 |
| 65 | Neophytadiene | C_20_H_38_ |  | 🗸 |  | 10446 |
| 66 | 9-Hexadecenoic acid, eicosyl ester, (Z)- (Arachidyl palmitoleate) | C_36_H_70_O_2_ |  | 🗸 |  | 5365040 |
| 67 | 11-Hexadecenoic acid, methyl ester | C_17_H_32_O_2_ |  | 🗸 | 🗸 | 5364696 |
| 68 | 1,2-Benzenedicarboxylic acid, butyl octyl ester (Butyl octyl phthalate) | C_20_H_30_O_4_ |  | 🗸 | 🗸 | 66540 |
| 69 | Linoleic acid ethyl ester (Ethyl Linoleate) | C_20_H_36_O_2_ |  | 🗸 | 🗸 | 5282184 |
| 70 | 16-Octadecenoic acid, methyl ester | C_19_H_36_O_2_ |  | 🗸 | 🗸 | 5364513 |
| 71 | Methyl oleate | C_19_H_36_O_2_ |  | 🗸 | 🗸 | 5364509 |
| 72 | N-Retinoyl DL-phenylalanine (all-trans) (2-[[(2E,4E,6E,8E)-3,7-dimethyl-9-(2,6,6-trimethylcyclohexen-1-yl)nona-2,4,6,8-tetraenoyl]amino]-3-phenylpropanoic acid) | C_29_H_37_NO_3_ |  | 🗸 |  | 5374060 |
| 73 | Heptadecanoic acid, 9-methyl-, methyl ester (Methyl 9-methylheptadecanoate) | C_19_H_38_O_2_ |  | 🗸 | 🗸 | 554038 |
| 74 | Methyl stearate | C_19_H_38_O_2_ |  | 🗸 | 🗸 | 8201 |
| 75 | Perhydroindene-4-carboxylic acid, 6-acetoxy-2,3-epoxy-1,1-epoxymethyl-3a-hydroxy-5-isopropenyl-7a-methyl-7-oxo-, methyl ester | C_18_H_22_O_8_ |  | 🗸 |  | 539690 |
| 76 | Cholestan-3-ol, 2-methylene-, (3.beta.,5.alpha.)-(5alpha-Cholestan-3beta-ol, 2-methylene-) | C_28_H_48_O |  | 🗸 |  | 22213932 |
| 77 | Incensole oxide | C_20_H_34_O_3_ |  | 🗸 |  | 90470329 |
| 78 | 4,4-Difluororetinol (all-trans) | C_20_H_28_F_2_O |  | 🗸 |  | 5366072 |
| 79 | [9-(Acetyloxy)-4a,7b,9a-trihydroxy-1,1,6,8-tetramethyl-5-oxo-1a,1b,4,4a,5,7a,7b,8,9,9a-decahydro-1H-cyclopropa[3,4]benzo[1,2-E]azulen-3-yl]methyl acetate | C_24_H_32_O_8_ |  | 🗸 |  | 538172 |
| 80 | 7,8,12-Tri-O-acetyl ingol | C_26_H_36_O_9_ |  | 🗸 |  | 536448 |
| 81 | 3Beta-chloro-5alpha-cholestane-5,6beta-diol 6-acetate | C_29_H_49_ClO_3_ |  | 🗸 | 🗸 | 14101545 |
| 82 | 10aH-2,12a-Methano-1H,4H-cyclopropa[5,6][1,3]dioxolo[2',3']cyclopenta[1',2':9,10]cyclodeca[1,2-d][1,3]dioxin-15-ol, 1a,2,7a,13,14,14a-hexahydro-1,1,6,6,9,9,11,13-octamethyl- , [1aR-(1aα,2α,7aα,7bS*,10aα,12aα,13α,14aα,15R*)]- | C_26_H_38_O_5_ |  | 🗸 |  | 101288169 |
| 83 | Cholestane-3,5-diol, 5-acetate, (3β,5α)- ([(3*S*,5*R*,8*S*,9*S*,10*R*,13*R*,14*S*,17*R*)-3-hydroxy-10,13-dimethyl-17-[(2*R*)-6-methylheptan-2-yl]-1,2,3,4,6,7,8,9,11,12,14,15,16,17-tetradecahydrocyclopenta[a]phenanthren-5-yl] acetate) | C_29_H_50_O_3_ |  | 🗸 |  | 91691425 |
| 84 | Bufa-20,22-dienolide, 14,15-epoxy-3,16-dihydroxy-, (3β,5β,15β,16β)- (3beta,16beta-dihydroxy-14beta,15beta-epoxy-5beta-bufa-20,22-dienolide) | C_24_H_32_O_5_ |  | 🗸 |  | 19937 |
| 85 | 9,11,18-Trihydroxy-6,18-epoxypimara-5,8(14),15-trien-7-one, 2Ac derivative | C_24_H_30_O_7_ |  | 🗸 |  | - |
| 86 | 3-Desoxo-3,16-dihydroxy-12-desoxyphorbol 3,13,16,20-tetraacetate | C_28_H_38_O_10_ |  | 🗸 |  | 537633 |
| 87 | Olean-12-ene-3,15,16,21,22,28-hexol, (3.β.,15.α.,16.α.,21.β.,22.α.)- (Olean-12-ene-3.beta.,15.alpha.,16.alpha.,21.beta.,22.alpha.,28-hexol) | C_30_H_50_O_6_ |  | 🗸 |  | 619407 |
| 88 | γ-Sitosterol (24-Ethylcholest-5-en-3beta-ol) | C_29_H_50_O |  | 🗸 |  | 457801 |
| 89 | 9,19-Cyclolanostane-3,7-diol | C_30_H_52_O_2_ |  | 🗸 |  | 634325 |
| 90 | Glycodeoxycholic acid | C_26_H_43_NO_5_ |  | 🗸 |  | 3035026 |
| 91 | Methyl 5,6-diacetyloxy-10-hydroxy-2,4b,7,7,10a,12a-hexamethyl-12-methylidene-1,4,8-trioxo-4a,5,6,6a,9,10,10b,~~g~~11-octahydronaphtho[1,2-h]isochromene-2-carboxylate | C_30_H_40_O_11_ |  | 🗸 |  | 45782844 |
| 92 | 2(4H)-Benzofuranone, 5,6,7,7a-tetrahydro-4,4,7a-trimethyl-, (7aR)- (Dihydroactinidiolide) | C_11_H_16_O_2_ |  |  | 🗸 | 6432173 |
| 93 | 1-(2-[3-(2-Acetyloxiran-2-yl)-1,1-dimethylpropyl]cycloprop-2-enyl)ethanone | C_14_H_20_O_3_ |  |  | 🗸 | 540318 |
| 94 | 4,6,10,10-Tetramethyl-5-oxatricyclo[4.4.0.0(1,4)]dec-2-en-7-ol | C_13_H_20_O_2_ |  |  | 🗸 | 538964 |
| 95 | 1-Nonadecene | C_19_H_38_ |  |  | 🗸 | 29075 |
| 96 | Tetradecane, 2,6,10-trimethyl- | C_17_H_36_ |  |  | 🗸 | 85785 |
| 97 | Heptadecane | C_17_H_36_ |  |  | 🗸 | 12398 |
| 98 | 3-Hydroxy-1a,5-bis(hydroxymethyl)-5,6b-dimethyl-1,3,3a,4,6,6a-hexahydrocyclopropa[e]inden-2-one | C_14_H_22_O_4_ |  |  | 🗸 | 45360340 |
| 99 | 9-Hexadecen-1-ol, (Z)- ((Z)-Hexadec-9-en-1-ol) | C_16_H_32_O |  |  | 🗸 | 5367661 |
| 100 | 9,12-Octadecadienoic acid, methyl ester, (E,E)- (Methyl linolelaidate) | C_19_H_34_O_2_ |  |  | 🗸 | 5362793 |
| 101 | Cis-13-Octadecenoic acid, methyl ester | C_19_H_36_O_2_ |  |  | 🗸 | 12541027 |
| 102 | trans-13-Octadecenoic acid, methyl ester (13-Octadecenoic acid, methyl ester) | C_19_H_36_O_2_ |  |  | 🗸 | 5364506 |
| 103 | Phytol | C_20_H_40_O |  |  | 🗸 | 5280435 |
| 104 | 10,13-Eicosadienoic acid, methyl ester (Methyl eicosa-10,13-dienoate) | C_21_H_38_O_2_ |  |  | 🗸 | 5365687 |
| 105 | Hexadecanoic acid, 2-hydroxy-1-(hydroxymethyl)ethyl ester (2-Palmitoylglycerol) | C_19_H_38_O_4_ |  |  | 🗸 | 123409 |
| 106 | (22S)-21-Acetoxy-6α,11β-dihydroxy-16α,17α-propylmethylenedioxypregna-1,4-diene-3,20-dione | C_27_H_36_O_8_ |  |  | 🗸 | 544325 |
| 107 | Stigmast-5-en-3-ol, oleate | C_47_H_82_O_2_ |  |  | 🗸 | 20831071 |
| 108 | Cholesterol | C_27_H_46_O |  |  | 🗸 | 5997 |
| 109 | 5-Chloro-6beta-nitro-5alpha-cholestan-3-one | C_27_H_44_ClNO_3_ |  |  | 🗸 | 22296655 |
| 110 | Cholestan-3-ol, 5-chloro-6-nitro-, (3β,5α,6β)- (Cholestan-3-ol, 5-chloro-6-nitro-, (3beta,5alpha,6beta)-) | C_27_H_46_ClNO_3_ |  |  | 🗸 | 22212711 |
| 111 | Octadecanoic acid, 10-oxo-, methyl ester (Methyl 10-oxooctadecanoate) | C_19_H_36_O_3_ |  |  | 🗸 | 543603 |
| 112 | Triarachidin | C_63_H_122_O_6_ |  |  | 🗸 | 522017 |
