# Supplementary figures and images for: Seaweed-derived bioactives with anti-tyrosinase activity: a potential for skin-whitening cosmetics with in silico and in vitro approaches
Source: Biotechnol Rep (Amst). 2025 Aug 7;47:e00910. doi: 10.1016/j.btre.2025.e00910 (PMC12357141; doi:10.1016/j.btre.2025.e00910)

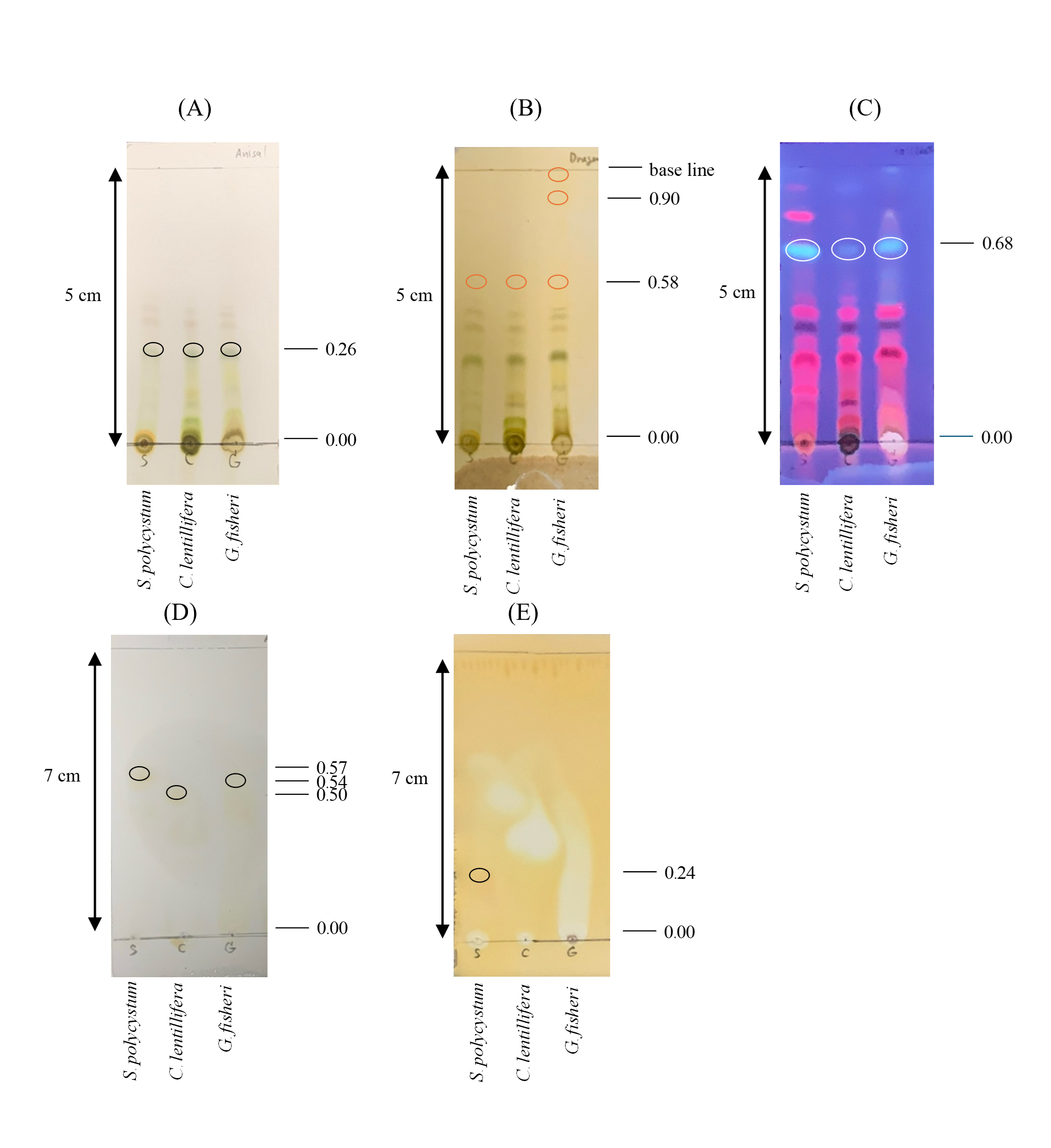

Supplement: Supplementary file 2 [file mmc2.zip › Supplementary Fig 1.tif]

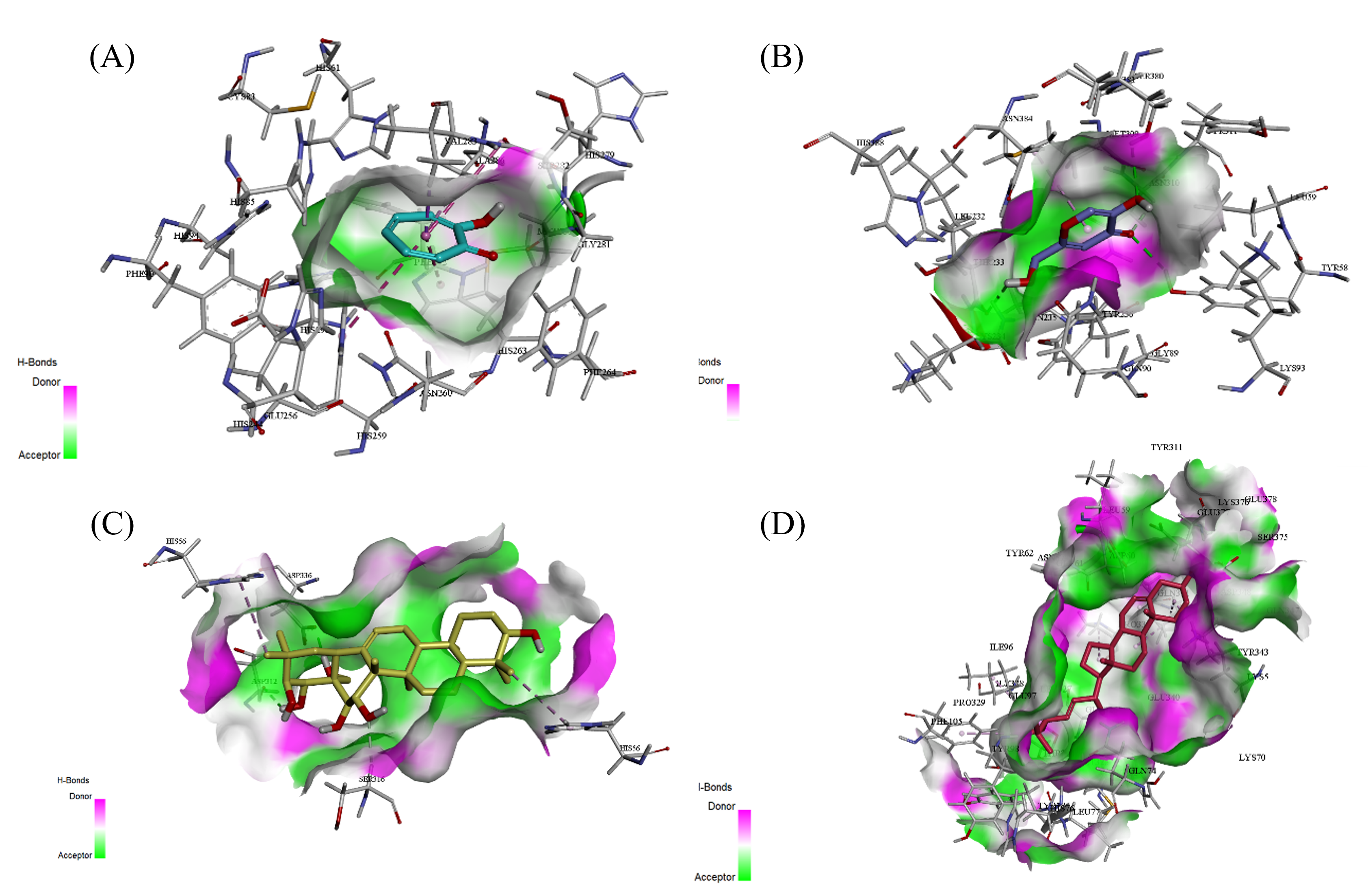

Supplement: Supplementary file 3 [file mmc3.zip › Supplementary Fig 2.tif]

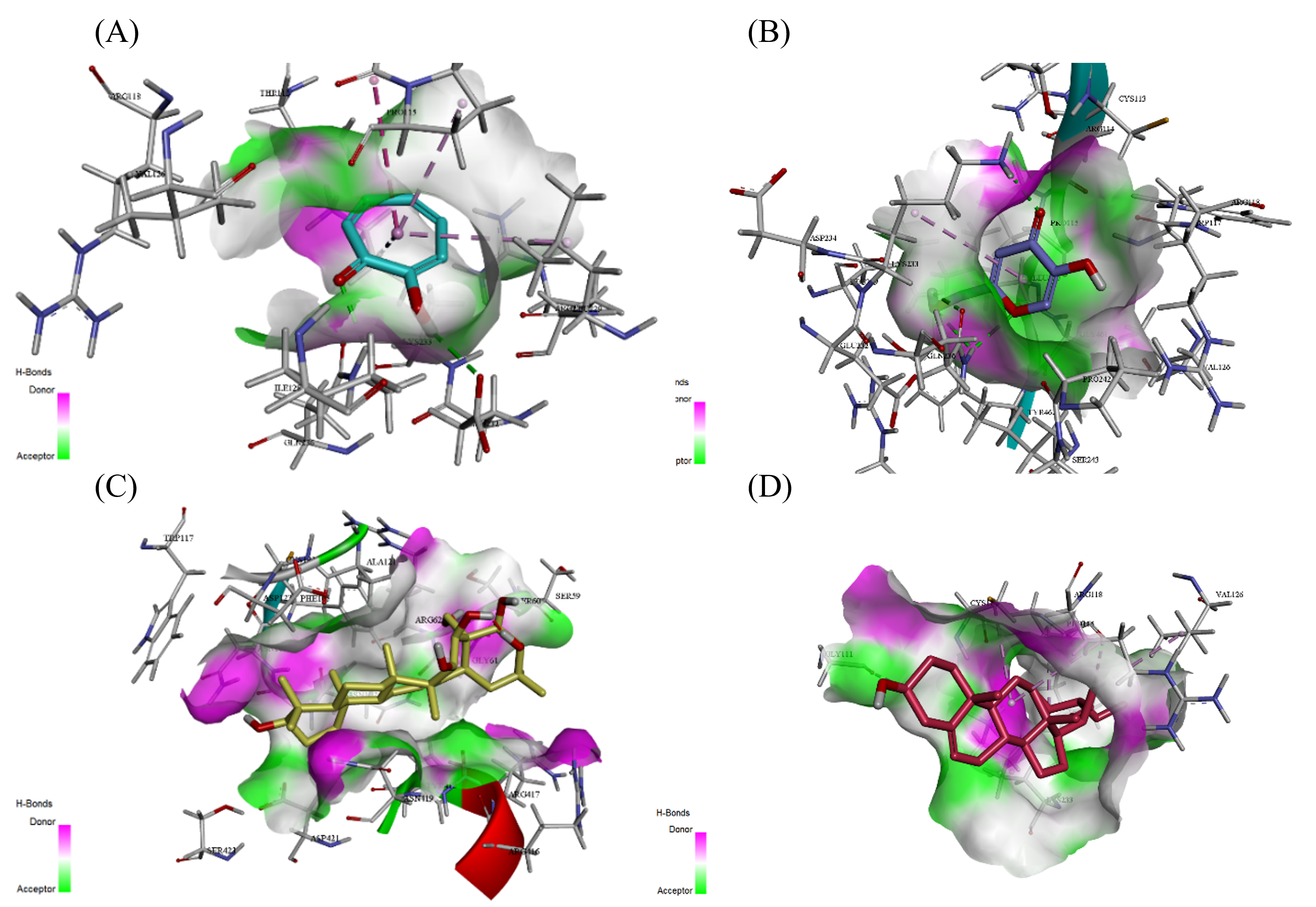

Supplement: Supplementary file 4 [file mmc4.zip › Supplementary Fig 3.tif]
